# A Kinome RNAi Screen Identified AMPK as Promoting Poxvirus Entry through the Control of Actin Dynamics

**DOI:** 10.1371/journal.ppat.1000954

**Published:** 2010-06-17

**Authors:** Theresa S. Moser, Russell G. Jones, Craig B. Thompson, Carolyn B. Coyne, Sara Cherry

**Affiliations:** 1 Department of Microbiology, Penn Genome Frontiers Institute, The University of Pennsylvania School of Medicine, Philadelphia, Pennsylvania, United States of America; 2 Goodman Cancer Center and Department of Physiology, McGill University, Montreal, Quebec, Canada; 3 Department of Cancer Biology, The University of Pennsylvania School of Medicine, Philadelphia, Pennsylvania, United States of America; 4 Department of Microbiology and Molecular Genetics, The University of Pittsburgh, Pittsburgh, Pennsylvania, United States of America; University of Alberta, Canada

## Abstract

Poxviruses include medically important human pathogens, yet little is known about the specific cellular factors essential for their replication. To identify genes essential for poxvirus infection, we used high-throughput RNA interference to screen the *Drosophila* kinome for factors required for vaccinia infection. We identified seven genes including the three subunits of AMPK as promoting vaccinia infection. AMPK not only facilitated infection in insect cells, but also in mammalian cells. Moreover, we found that AMPK is required for macropinocytosis, a major endocytic entry pathway for vaccinia. Furthermore, we show that AMPK contributes to other virus-independent actin-dependent processes including lamellipodia formation and wound healing, independent of the known AMPK activators LKB1 and CaMKK. Therefore, AMPK plays a highly conserved role in poxvirus infection and actin dynamics independent of its role as an energy regulator.

## Author Summary

Entry is a vital step in establishing viral infection, providing a potential therapeutic target. Many viruses co-op one of the various cellular endocytic routes for entry, making the host factors that contribute to these processes essential for efficient infection. In particular, vaccinia, the prototypical poxvirus, takes advantage of macropinocytosis for efficient uptake. To identify the signaling factors required for entry of vaccinia virus we performed an RNAi screen of the Drosophila kinome for those kinases and phosphatases that facilitate infection. We identified seven genes including the three subunits of AMPK as promoting infection, and found that AMPK was also required in mammalian cells for vaccinia infection. Furthermore, we found that AMPK facilitates vaccinia entry thru its ability to modulate the actin cytoskeleton and macropinocytosis. In addition to promoting vaccinia uptake, we found that AMPK also contributes to other virus-independent but actin-dependent processes including lamellipodia formation and cellular motility, indicating a broad cellular role in regulating actin dynamics.

## Introduction

In order to successfully infect cells, viruses must remodel the cellular environment to allow for the reallocation of resources to viral production. Poxviruses are large double stranded (ds) DNA viruses that have a sophisticated lifecycle characterized by a number of temporally regulated steps. Vaccinia virus is the prototypical poxvirus, was used as the vaccine to eradicate smallpox, and has been the most thoroughly characterized [1]. To initiate infection, vaccinia first binds, enters cells, uncoats, and expresses early gene products. Next, genomic DNA replication occurs, followed by intermediate and late gene expression. Assembly, maturation, and virus release completes the cycle. Although poxviruses encode a large number of genes (>200), they remain obligate intracellular pathogens and require a multitude of activities hijacked from their host cell. While many viral factors required for various steps in the vaccinia lifecycle have been described, the specific host factor contribution is less clear.

In particular, an early step in the infection cycle involves cell penetration. This step is critical for the initial establishment of infection, and also presents a good target for anti-viral therapeutics [2]. Different families of viruses have developed diverse strategies for entering cells; some fuse at the plasma membrane, while others co-opt one of the many cellular endocytic routes [3]. Studies have demonstrated that macropinocytosis is an important endocytic route of vaccinia entry [4]. Generally, macropinocytosis is a nonselective route for bulk fluid-phase uptake and is not constitutively active, but is induced by growth factors, and also by some pathogens including vaccinia [5] [6]. This active endocytic process induces extensive actin cytoskeletal rearrangement, leading to membrane ruffling, lamellipodia formation, and the internalization of extracellular fluid and membrane. Consistent with this, vaccinia entry is dependent upon modulation of the actin cytoskeleton, and initiates macropinocytosis by inducing dramatic actin-rich microvilli protrusions followed by global myosin II-dependent blebbing, thereby promoting virus uptake [4] [7]. Induction triggers the activation of receptor tyrosine kinases (RTKs) which activate complex signaling cascades leading to the induction of these actin extensions which extend the plasma membrane allowing fluid-phase capture. This process involves signaling cascades that converge on members of the Ras superfamily of GTPases in particular, Rab5 and Rac1 [6] [8]. Rac1 contributes to a number of cellular processes that require extensive actin dynamics, and its signaling is carefully regulated by several guanine exchange factors as well as by crosstalk with other Rho family GTPases [9] [10] [11]. Again, as for growth factor dependent macropinocytosis, vaccinia-induced uptake is dependent upon Rac1 [4] [7]. Additional kinases such as p21-activated kinase (PAK1) are then activated along with actin-associated proteins that lead to large-scale actin rearrangements, lipid modifications, and ultimately macropinosome formation [4] [5]. While some specific kinase families have been implicated in macropinocytosis (e.g., protein kinase C (PKC), serine/threonine kinases, tyrosine kinases, and phosphatidylinositol kinases) [5], many of the specific factors have not been identified, and in some cases the specific role of factors such as PKC, is not well understood. Therefore, there are many additional cellular signaling factors remaining to be identified for this complicated uptake mechanism and thus, for vaccinia entry.

To take an unbiased systematic approach toward the identification of these cellular factors, we developed a system using the model organism *Drosophila* to perform a high-throughput RNA interference (RNAi) screen for cellular kinases and phosphatases required for early steps in vaccinia infection. The *Drosophila* system is particularly amenable to this approach for a number of reasons including: reduced redundancy in the genome, high conservation with mammalian systems, efficient RNAi, and previous success with this system to identify cellular factors required for viral infection [12] [13]. This *Drosophila* system is permissive to early steps in the vaccinia infection cycle allowing us to specifically dissect the role of cellular factors involved in the infectious entry process.

Using this system, we identified seven genes that contribute to vaccinia infection, including the three subunits of the AMP-activated kinase (AMPK) complex, the master energy sensor of the cell. Importantly, the requirement for AMPK in vaccinia infection is conserved in mammalian cells, and is specifically required for vaccinia-induced macropinocytic entry. Further characterization led to the discovery that AMPK controls a variety of virus-independent actin-dependent processes including lamellipodia formation and cell migration. Altogether, we found a new role for AMPK in actin dynamics.

## Results

### Vaccinia infection in *Drosophila* cells

**Figure 1:**
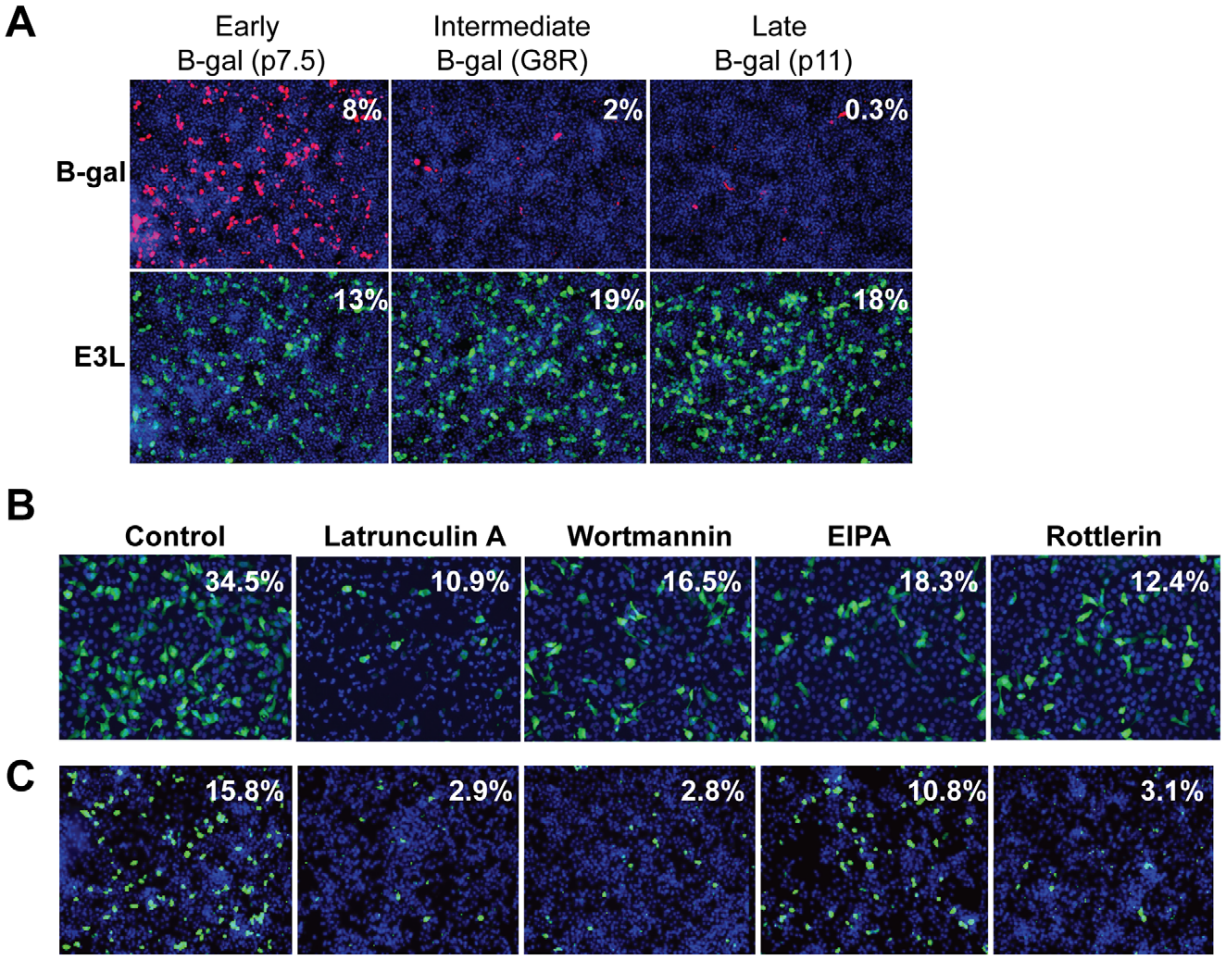
Vaccinia virus undergoes entry in *Drosophila* cells. **A**. *Drosophila* cells were infected with three different recombinant vaccinia viruses expressing B-gal driven by an early/late (p7.5), intermediate (G8R), or late (p11) vaccinia promoter. Each virus expresses E3L protein from an endogenous early promoter. After 48 hours, infection levels were assessed using B-gal or E3L-specific antibodies. **B–C**. Vaccinia infection is dependent upon known mediators of macropinocytosis. **B**. Human U2OS cells were pretreated with: Latrunculin A, Wortmannin, and Rottlerin at 5 µM; EIPA at 12.5 µM for 1 hour and challenged with vaccinia (MOI = 10) for 8 hours. **C**. *Drosophila* DL1 cells were treated with: Latrunculin A, Wortmannin, and Rottlerin at 5 µM; EIPA at 50 µM for 1 hour and challenged with vaccinia (MOI = 20) for 24 hours. Cells were fixed and processed for immunofluorescence using E3L expression (green) as a marker for infection, and Hoescht 33342 (blue) to visualize nuclei. Percent infection is the average of four images in a representative of three experiments.

Since vaccinia infection of *Drosophila* cells has not been reported, we first characterized the course of infection in *Drosophila* cells. Using a reporter virus expressing Beta-galactosidase (B-gal) under the control of an early/late promoter which is active during all stages of vaccinia infection, we found that vaccinia infection is dose-dependent with maximal expression at 48 hours post infection (hpi) (Figure S1). Next, we infected *Drosophila* cells using reporter viruses that express B-gal under the control of temporally regulated vaccinia promoters that are active during different phases of the virus replication cycle. We found that *Drosophila* cells were efficiently infected as measured by the production of B-gal from an early/late promoter (p 7.5) or by the production of E3L protein, a vaccinia gene product expressed early in infection, while there was very little expression of B-gal from either an intermediate promoter (G8R), or a late promoter (p11) (Figure 1A). Consistent with these findings, we have been unable to detect vaccinia DNA replication (data not shown) suggesting a block to infection following early protein synthesis. These findings demonstrate that while vaccinia is unable to complete all stages of the lifecycle in *Drosophila* cells, entry and early expression occur, providing a model system to study the host factor requirements of vaccinia entry.

Previous studies have shown that efficient vaccinia entry is dependent upon the endocytic route of macropinocytosis [4]. In order to assess whether host requirements for vaccinia entry were conserved between *Drosophila* and mammalian cell lines, we tested whether inhibition of macropinocytosis attenuated infection in these disparate cell types. To this end, we treated cells with several known inhibitors of macropinocytosis and vaccinia entry including; an actin inhibitor Latrunculin A, phosphoinositide-3-kinase (PI3K) inhibitor Wortmannin, Na/H antiporter inhibitor EIPA, and PKC inhibitor Rottlerin [4] [7]. We found that each of these drugs significantly inhibited vaccinia infection in both human and *Drosophila* cells (Figure 1B–C, quantified in Figure S2). These data show that at least early steps in the viral lifecycle are dependent upon similar pathways in insect cells allowing us to use this model to identify additional factors required for vaccinia infection in mammalian cells.

### RNAi screen of *Drosophila* kinome

**Figure 2:**
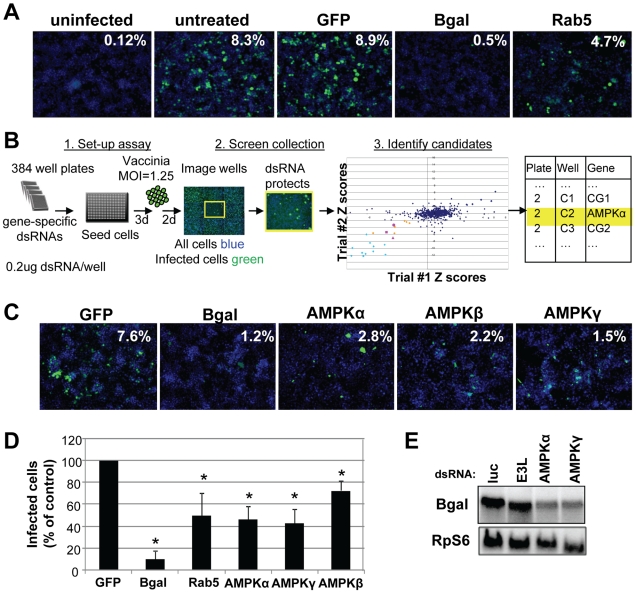
High-content RNAi screen identifies cellular factors required for vaccinia infection. **A**. Cells were pre-treated with the indicated dsRNAs and infected with E/L (p7.5) B-gal vaccinia at MOI of 1.25 for 48 hours. Percentage of infected cells is calculated from ((B-gal^+^, green)/(nuclei, blue)). **B**. Schematic of high-throughput RNAi screening. Cells were plated in 384 well plates arrayed with gene-specific dsRNA targeted against the *Drosophila* kinome. After three days, the cells were infected with E/L B-gal vaccinia at MOI = 1.25 for two days and then processed for immunofluorescence. Automated image analysis was used to determine the percentage of infected cells and used to identify the positive candidates (Z<−2 in duplicate). Candidates in light blue represent the B-gal-depleted positive controls, and those in pink represent the three subunits of the heterotrimeric AMPK complex. Other candidates are indicated in orange. **C**. Examples of primary screen data including AMPK components that when depleted resulted in a significant decrease in the percentage of B-gal-positive cells. **D**. Independent dsRNAs against each of the three AMPK subunits: AMPKα (SNF1A), AMPKβ (alicorn) and AMPKγ (SNF4Agamma). RNAi was performed and cells were infected at an MOI = 5. Percent infection was measured and the mean +SD is shown; * indicates p<0.05 compared to control in three independent experiments. **E**. Northern blot analysis was performed on RNA extracted from cell lysates pretreated with dsRNA targeting the indicated genes and probed for B-gal, and a loading control RpS6.

In order to systematically probe the requirements for cellular signaling factors in early vaccinia infection, we developed a quantitative assay amenable to RNAi using virally encoded B-gal expression as a measure of early infection. While a non-targeting negative control dsRNA (GFP) had no effect on the percentage of infected cells, knock-down of B-gal by RNAi reduced the percentage of B-gal expressing cells 17-fold, indicating that vaccinia infection can be quantitatively assayed using this system (Figure 2A). Moreover, dsRNA targeting the cellular gene Rab5, a small GTPase required for many endocytic processes including macropinocytosis [8] also significantly decreased vaccinia infection, validating that we can identify cell-encoded factors required for vaccinia infection using this approach (Figure 2A and 2D).

We used this assay to perform an RNAi screen against the *Drosophila* kinome to identify novel signaling factors that promote vaccinia infection (Schematic diagram Figure 2B). This screen consisted of approximately 440 unique genes (∼200 kinases, ∼90 phosphatases, and ∼150 regulator factors) arrayed onto 384 well plates (Table S1). Additionally, negative control wells were included containing either no dsRNA (15 wells) or dsRNA targeting GFP (28 wells), which is not expressed in this system. Lastly, 21 positive control wells with dsRNA targeting *lacZ* were included (Figure 2B light blue). *Drosophila* cells were seeded in these pre-arrayed 384 well plates, incubated for three days to allow knock down of each targeted gene, and then infected with vaccinia virus for 48 hours. For the screen, a baseline infection of 10% was within the linear range and was achieved at a multiplicity of infection (MOI) of 1.25 (Figure S1C). The plates were fixed and processed for immunofluorescence using B-gal expression as a measure of infection, and counter-stained to monitor cell number. Automated microscopy and image analysis were used to quantify the percent infection (B-gal^+^/Total Nuclei) that was transformed into Robust Z scores for each plate, and the Robust Z scores of the 2 replicates were plotted against each other (Figure 2B). Positive candidates were defined as having a Robust Z score of <−2 in duplicate screens (p<0.05). Using these metrics, we identified 8 genes (2%, orange and pink Figure 2B). In addition to these 8 factors, we identified 20 out of 21 (95%) of the positive control *lacZ* dsRNAs (Figure 2B light blue and Table S1) and none of the 43 negative controls (non-targeting dsRNA and empty wells). We also monitored the toxicity of the dsRNA treatments and found that none of the 8 genes that inhibited infection were cytotoxic (<25% decrease in cell number, Table 1). In contrast, we found that while 17 wells reduced cell number by >25% in duplicate screens, none of these genes also inhibited infection. Therefore, our screen revealed host factors required for robust infection that are not required for cell viability. Notably all 8 genes have human homologs (Table 1), including all 3 subunits of the AMP-activated kinase (AMPK) complex (SNF1A (AMPKα), SNF4Agamma (AMPKγ) and alicorn (AMPKβ)) (Figure 2B pink), a heterotrimeric complex involved in maintaining cellular energy homeostasis. RNAi resulted in ∼3-fold reduction in vaccinia infection when AMPK was depleted (Figure 2C).

**Table 1:** Candidate genes identified and validated.

		% infection		Robust Z score		Nuclei fold change		Validated?
*Drosophila* Gene	Human Gene	Screen #1	Screen #2	Screen #1	Screen #2	Screen #1	Screen #2	
median		3.9	7			1	1	
B-gal		0.9	1.2	−17.5	−10.8	1	1	
SNF1A	PRKAA2	1.7	2.8	−4.5	−5.4	0.8	1	Yes
SNF4Agamma	PRKAG2	1.1	1.9	−3.9	−7	1	0.9	Yes
alicorn	PRKAB1	0.3	4.2	−3.9	−3.4	1	1.1	Yes
Fab1	PIKfyve	3.4	4.5	−2.3	−2.2	1	1	Yes
Pi3K68D	PIK3C2A	2.4	3.3	−3.8	−3.9	1.1	1.1	Yes
Stam	STAM	3.3	4.5	−2.1	−2.5	1	1.1	Yes
CG9311	PTPN23	3.1	2.5	−3.8	−5.7	1	0.9	Yes
Strn-Mlck	MYLK	0.6	4.8	−2.5	−2.8	1	1.1	No

Non-overlapping secondary dsRNAs were generated for each of the 8 genes and were tested to confirm the role of each of these genes in vaccinia infection. Seven of the genes validated (88%). Importantly, among the genes that validated were those encoding the three subunits of the AMPK complex (Figure 2D, Figure S3 and Table 1). Moreover, RT-PCR confirmed that AMPKα was depleted by dsRNA treatment against AMPKα (Figure S4A). Furthermore, we found that loss of AMPKα or AMPKγ also led to a defect in early viral mRNA accumulation compared to control (Figure 2E), suggesting that AMPK is required upstream of viral mRNA production in *Drosophila*, perhaps at the stage of entry.

### AMPK promotes vaccinia infection in mammalian cells

AMPK is an important sensor of intracellular energy that is conserved in eukaryotes ranging from yeast to humans [14]. While *Drosophila* encodes only one copy of each AMPK subunit, mammals have multiple isoforms of each subunit encoded by several distinct genes (α1, α2, β1, β2, γ1, γ2, γ3) which can produce at least 12 possible heterotrimeric combinations [15]. The lack of redundancy in *Drosophila* allowed our identification of AMPK by single gene RNAi. We used this *Drosophila* system as a tool to identify novel host factors that contribute to vaccinia infection, but since *Drosophila* is not a natural host, we were interested in determining the role of AMPK in a more biologically relevant context.

**Figure 3:**
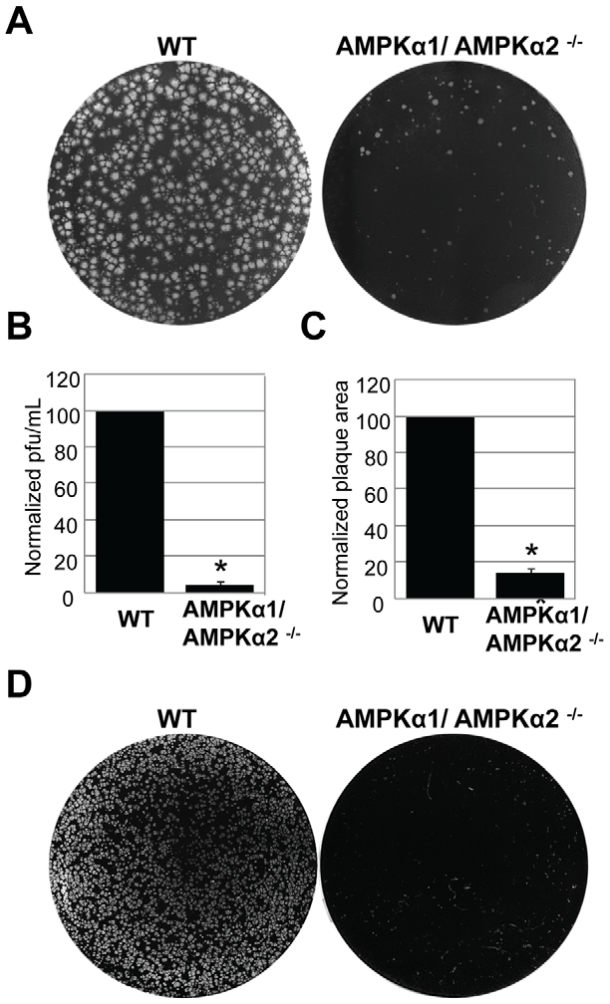
AMPKα promotes poxvirus infection in mammalian cells. **A**. Vaccinia virus plaque assays were performed with wild type or AMPKα1/AMPKα2 ^−/−^ MEFs. Representative data from the10^−5^ dilution of virus is shown. **B**. Quantification of plaques from **A**. presented as the normalized mean +SD of wild type plaques from four experiments;* indicates p<0.05. **C**. The diameter of 30 representative plaques in each duplicate well from **A**. was used to calculate the average plaque area, which is displayed as the normalized mean +SD in triplicate experiments; * indicates p<0.05. **D**. Cowpox virus plaque assays were performed with wild type or AMPKα1/AMPKα2 ^−/−^ MEFs. Representative data from the10^−5^ dilution of virus is shown.

**Figure 4:**
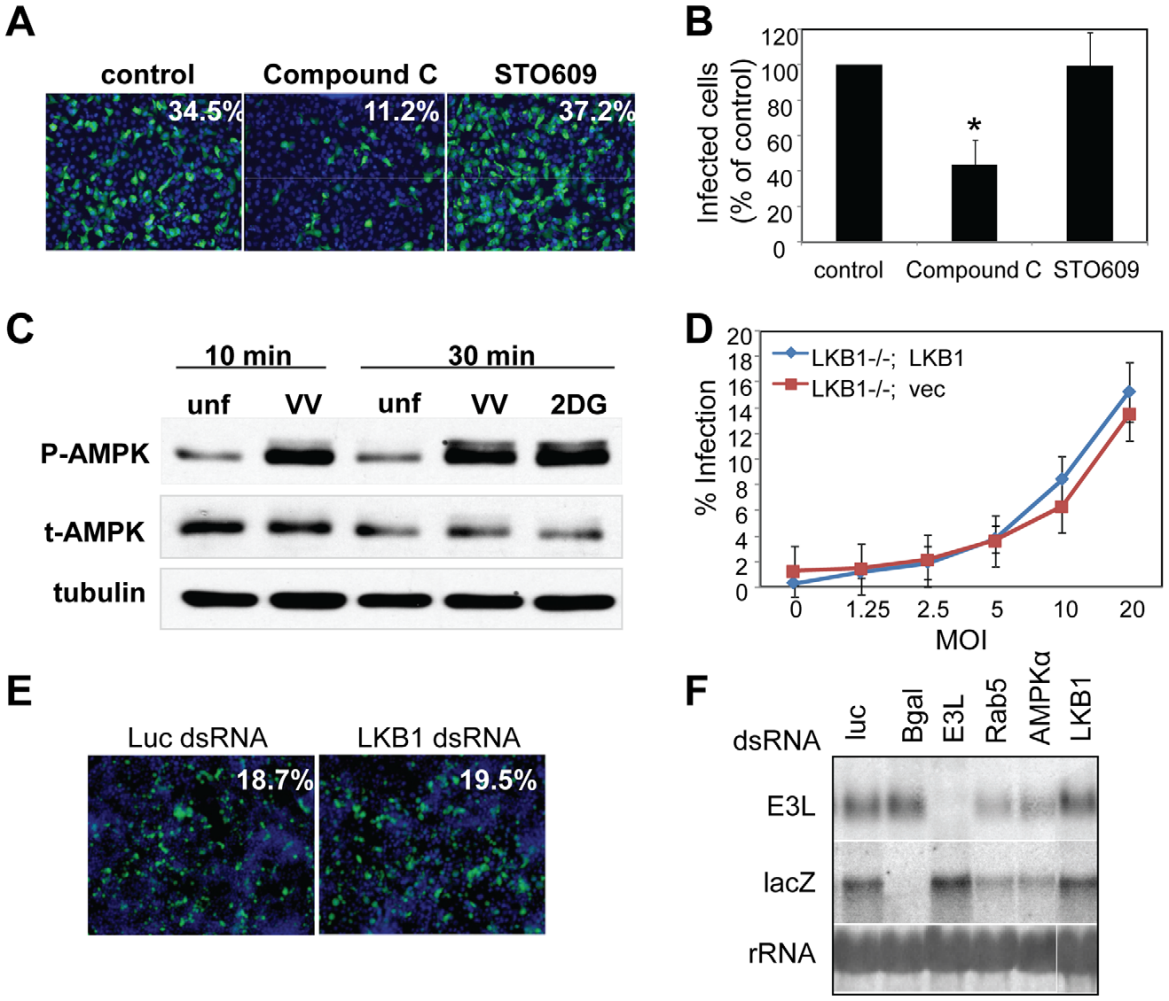
Vaccinia infection induces AMPK activation, and is independent of LKB1 and CaMKK. **A**. Human U2OS cells were pre-treated with vehicle, Compound C (1–5 µM), or STO609 (1–5 µg/mL) and then infected with vaccinia (MOI = 10) for 8 hours (E3L, green; nuclei, blue). A representative of 3 experiments is shown. **B**. Percent infection of **A** was measured and the mean +SD is shown; * indicates p<0.05 compared to control in three independent experiments. **C**. Cells were pretreated with vaccinia virus (MOI = 20) at 16°C for 1 hour, and then infected at 37°C or treated with 2DG, a known inducer of AMPK phosphorylation. Lysates were collected at the indicated time points post treatment and assayed by immunoblot for phospho-AMPK. A representative of 3 experiments is shown. **D**. LKB1^−/−^ MEFs were complemented with a vector control (LKB1^−/−^; Vec) or FLAG-LKB1 cDNA (LKB1^−/−^; LKB1) and subsequently infected with vaccinia at the indicated MOI for 8 hours, and processed for immunofluorescence. Percent infection was quantified using automated image analysis. A representative of two experiments is shown. **E**. RNAi against LKB1 in *Drosophila* cells had no effect on infectivity compared to RNAi against negative control luciferase as measured by immunofluorescence. **F**. LKB1 knock down in *Drosophila* has no effect on vaccinia early mRNA levels as measured by Northern blot. Cells were pretreated with dsRNAs against negative control luciferase, positive controls E3L (viral) and Rab5 (cellular), as well as AMPKα and LKB1 and probed as indicated.

To investigate the role of AMPK in vaccinia virus infection of mammalian cells, we took advantage of mouse embryonic fibroblasts (MEFs) that are genetically altered and null for both AMPKα subunits, AMPKα1 and AMPKα2 (AMPKα1/AMPKα2 ^−/−^) and verified the lack of these proteins by immunoblot analyses (Figure S5) [16] [17] [18]. These cell lines divide and grow indistinguishably from their sibling control AMPKα1/AMPKα2 ^+/+^ cells (wild type) (data not shown). We challenged either the AMPKα1/AMPKα2^−/−^ cells or their sibling control wild type cells with vaccinia virus and measured infection using a plaque assay. This revealed a 20-fold decrease in plaque number and a 15-fold decrease in plaque area in AMPKα1/AMPKα2 ^−/−^ compared to wild type cells (Figure 3A–C). The requirement for AMPK was also observed for the closely related poxvirus, cowpox virus (Figure 3D, quantified in Figure S6). These decreases in infectivity were specific for poxviruses and not simply due to a decrease in overall cell health since several unrelated RNA viruses, including Vesicular Stomatitis virus (VSV) grew as well in the AMPK deficient MEFs compared to wild type (Figure S7 and data not shown). This suggests that AMPK deficient cells are capable of supporting all stages of virus infection; including at least some forms of endocytosis and endosomal trafficking since VSV enters cells through clathrin-mediated endocytosis [19]. Therefore, we identified a specific requirement for AMPK in poxvirus infection but not for viral infection generally. In addition to plaque assays we also monitored vaccinia infection by immunofluorescence, immunoblot, and Northern blot and found that there was a significant decrease in vaccinia virus replication in AMPKα1/AMPKα2 ^−/−^ cells in each assay (Figure S8A–C). This suggests that AMPK promotes early steps of the vaccinia lifecycle in mammalian cells as well as *Drosophila*. Finally, to verify that the requirement for AMPK was not MEF-specific we tested whether inhibition of AMPK in the human osteosarcoma cell line U2OS attenuated vaccinia infection using two approaches. First, we pre-treated U2OS cells with the AMPK inhibitor Compound C or vehicle and challenged these cells with vaccinia [20]. Again, we found that inhibition of AMPK attenuated infection (Figure 4A, B). Next, we depleted AMPKα1, AMPKα2, or both AMPKα1 and AMPKα2 using siRNAs and observed a decrease in vaccinia infection (Figure S9A, B, Text S1). We confirmed knock-down by immunoblot (Figure S9C). Together, these data show that vaccinia infection is dependent upon AMPK for infection across disparate cell types including *Drosophila*, human and mouse.

### Vaccinia infection activates AMPK

AMPK is activated through phosphorylation of a threonine residue on the catalytic α subunit, which can be triggered by a variety of stimuli including an increase in the cellular ratio of AMP/ATP [21] [22] [23] [24] [25] [26]. Since AMPK promotes vaccinia infection, we tested whether infection activates AMPK. We used a phospho-specific antibody against AMPKα to measure AMPK activation. Treatment with 2-deoxyglucose (2DG), a known activator of AMPK, led to an increase in AMPK phosphorylation, while little phosphorylation was detected in untreated controls (Figure 4C). Moreover, we observed an increase in phospho-AMPKα within 10 minutes of vaccinia infection (Figure 4C). This increase was not due to changes in total AMPK levels, and suggests that AMPK becomes activated very early in vaccinia infection.

### Known upstream activators of AMPK are not required for vaccinia infection

Several upstream kinases have been implicated in AMPK activation under different conditions. The classic activator of AMPK is the tumor suppressor LKB1, which activates AMPK in response to energy deprivation [27] [28]. In *Drosophila*, LKB1 is the only described upstream kinase required for AMPK activation and *lkb1* mutants phenocopy *ampk* mutants [29] [30]. In contrast, in mammalian systems, LKB1 is the upstream kinase in response to energy deprivation, while additional upstream kinases, such as calcium/calmodulin-dependent protein kinase kinase beta (CaMKKβ) have been implicated in AMPK activation under other conditions [31] [32]. We tested whether LKB1 was required for vaccinia infection using cells that are null for LKB1 [33] and complemented with either vector alone (LKB1^−/−^; Vec), or an LKB1 cDNA (LKB1^−/−^; LKB1) (Figure S10) and found that loss of LKB1 had no effect on vaccinia infection in mammalian cells (Figure 4D). Likewise, using RNAi to deplete LKB1 in *Drosophila* cells, we found that it was dispensable for infection by immunofluorescence (Figure 4E) and Northern blot (Figure 4F). RT-PCR analysis validated that LKB1 was indeed knocked down in *Drosophila* cells (Figure S4B). We also tested whether CaMKK, the other well-established AMPK activator in mammalian systems, was required for vaccinia infection. To this end, we pre-treated U2OS cells with the CaMKK inhibitor STO609 prior to infection, and found no effect on vaccinia infection with doses up to 5 µg/ml (Figure 4A, B). Taken together, these data show that vaccinia infection is AMPK-dependent but LKB1 and CaMKK-independent.

### AMPK promotes vaccinia entry

**Figure 5:**
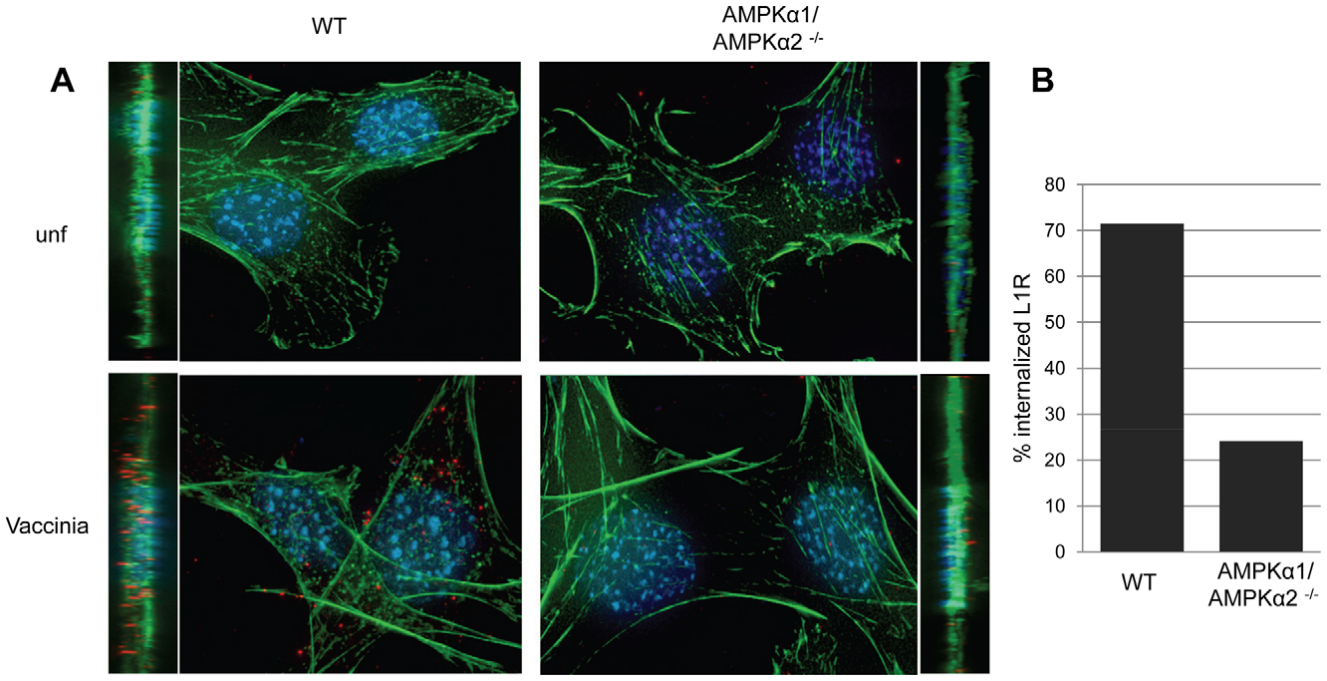
AMPK promotes vaccinia entry. **A**. Wild type or AMPKα1/AMPKα2 ^−/−^ MEFs were either infected or mock-infected with vaccinia, and the L1R membrane protein (red) was monitored to visualize incoming virus. Nuclei (blue) and actin (phalloidin (green)) were also stained. Images are presented as max projection along with XZ insets. Representative images of triplicate experiments are shown. **B**. The percentage of cells undergoing vaccinia entry was quantified (n>30 for each condition).

Given that loss of AMPK led to a decrease in both viral mRNA and protein production (Figure 2, Figure S8), we tested whether AMPK was required for efficient virus entry. We monitored viral entry into wild type or AMPKα1/AMPKα2 ^−/−^ MEFs using a fluorescence-based assay. We prebound virus to the cells, and then allowed infection to proceed for one hour. Incoming virus particles were visualized using an antibody against L1R, a membrane-bound viral surface protein (Figure 5A). Deconvolution of Z stacks was used to visualize vaccinia inside of cells (Figure 5A, XZ view). Quantification revealed a ∼3-fold reduction in the number of AMPK mutant cells that internalized virus (Figure 5B). These data show that AMPK promotes infection at the stage of entry, although we have not ruled out that virus binding could also be affected by lack of AMPK.

We were also interested in whether AMPK played a role in vaccinia infection downstream of entry. First, we monitored both early and late gene expression and found that while the percentage of cells expressing an early protein (E3L) or a late protein (L1R) was reduced in AMPKα1/AMPKα2 ^−/−^ MEFs compared to wild type, 100% of the cells that expressed early genes also expressed late genes, indicating no further block to replication (Figure S11A). We also measured the infectivity of virus produced from AMPKα1/AMPKα2 ^−/−^ MEFs. We found a ∼3-fold decrease in virus titer produced from AMPKα1/AMPKα2 ^−/−^ MEFs compared to wild type (Figure S11B) which is similar to the decrease in viral entry (Figure 5B). These data suggest that while fewer AMPK deficient cells produce virus, the vaccinia released from these cells is as infectious as virus produced from wild type cells. Therefore, while AMPK is important for entry, it is dispensable for later steps in the viral lifecycle.

### AMPK is required for vaccinia induced macropinocytosis

**Figure 6:**
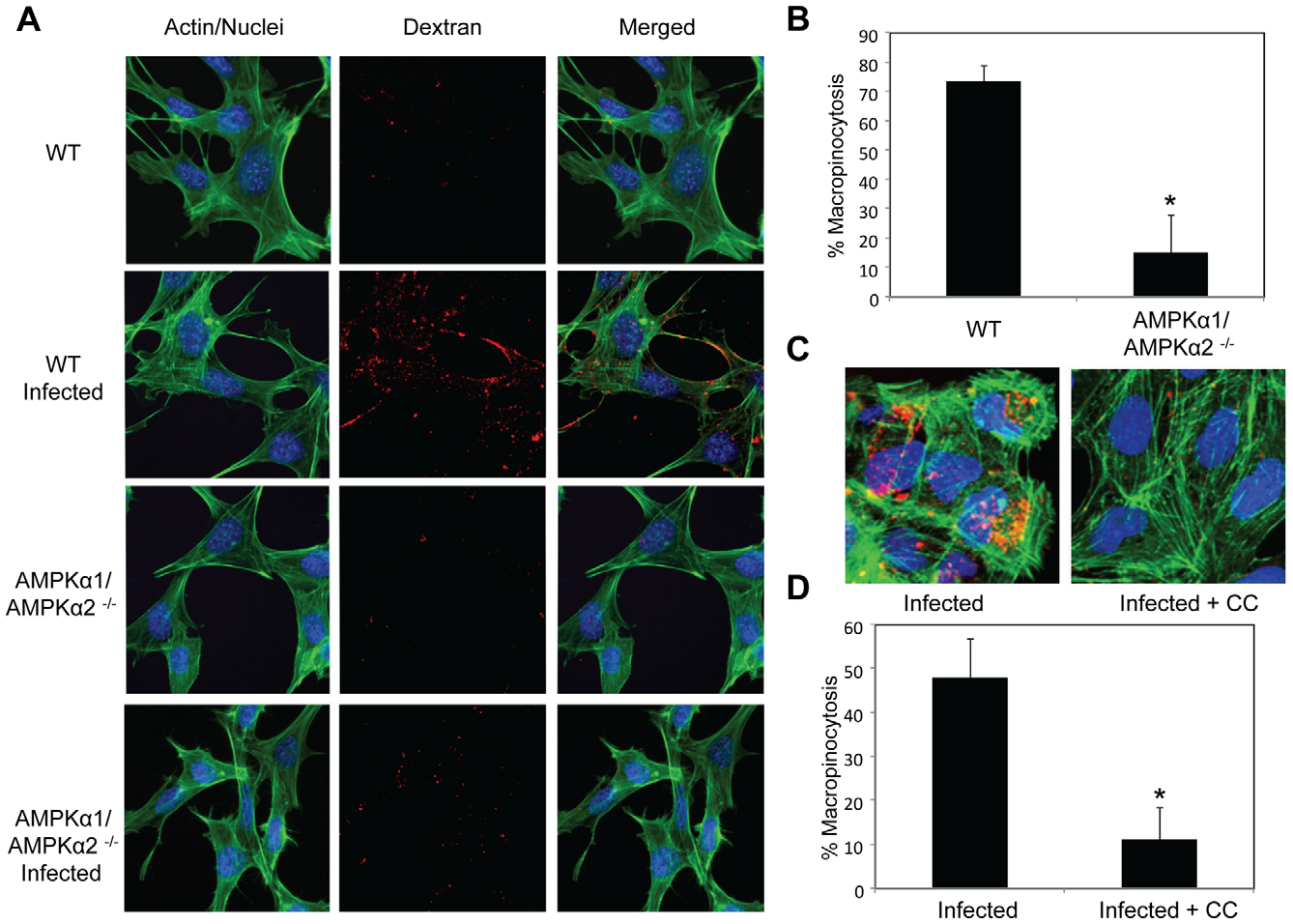
AMPK is required for vaccinia-induced macropinocytosis. Fluid phase dextran uptake assays were performed in the presence or absence of virus. **A**. Wild type and AMPKα1/AMPKα2 ^−/−^ MEFs were either infected or mock-infected and treated with FITC-dextran (red), processed for microscopy, and stained to visualize actin (phalloidin (green)) and nuclei (Hoescht 33342 (blue)). Representative images from triplicate experiments are shown. **B**. The percentage of MEFs with dextran punctae was quantified from three independent experiments with the mean + SD shown; * p<0.05. **C**. U2OS cells were treated or mock-treated with the AMPK inhibitor Compound C (10 µM) 1 hour prior to addition of vaccinia. Texas Red-dextran (red) was added and processed as above. **D**. The percentage of U2OS cells with dextran punctae was quantified from three independent experiments with the mean +/− SD shown; *p<0.05.

Previous studies established that a major entry route for vaccinia is macropinocytosis, which is required for and induced by vaccinia infection [4]. Since we observed a defect in viral entry in the AMPK mutant cells (Figure 5), and that inhibitors of macropinocytosis attenuated vaccinia infection (Figure 1), we tested whether AMPK plays a role in vaccinia-induced macropinocytosis. Macropinocytosis can be directly monitored by fluorescently labeled dextran uptake [34]. Neither wild type nor AMPKα1/AMPKα2 ^−/−^ MEFs efficiently endocytosed dextran under resting conditions (Figure 6A). However, upon vaccinia infection, macropinocytosis was dramatically induced in wild type MEFs as measured by an increase in dextran uptake into the cell. In contrast to the large number of dextran punctae observed in the infected wild type MEFs, AMPKα1/AMPKα2 ^−/−^ MEFs did not efficiently take up the dextran in the presence of virus (Figure 6A). We quantified the level of vaccinia-induced macropinocytosis in the wild type versus AMPK deficient cells and found an approximately five-fold reduction in the percentage of cells undergoing macropinocytosis (Figure 6B).

Furthermore, we found a decrease in vaccinia-induced dextran uptake in human U2OS cells pretreated with Compound C compared to vehicle control (Figure 6C). Quantification revealed an approximately five-fold decrease in the percentage of U2OS cells undergoing vaccinia-induced macropinocytosis when AMPK is inhibited (Figure 6D). Together, these data show that virus-induced macropinocytosis is dependent upon AMPK in disparate cell types and hosts.

In contrast to this dependence of macropinocytosis on AMPK, there was no defect in transferrin uptake in AMPKα1/AMPKα2 ^−/−^ MEFs (Figure S12, Text S1), indicating that receptor-mediated endocytosis is not controlled by AMPK. This is consistent with our observation that VSV infection is not attenuated in AMPKα1/AMPKα2 ^−/−^ MEFs as VSV enters cells by receptor-mediated endocytosis and not macropinocytosis (Figure S7).

### AMPK promotes lamellipodia formation

**Figure 7:**
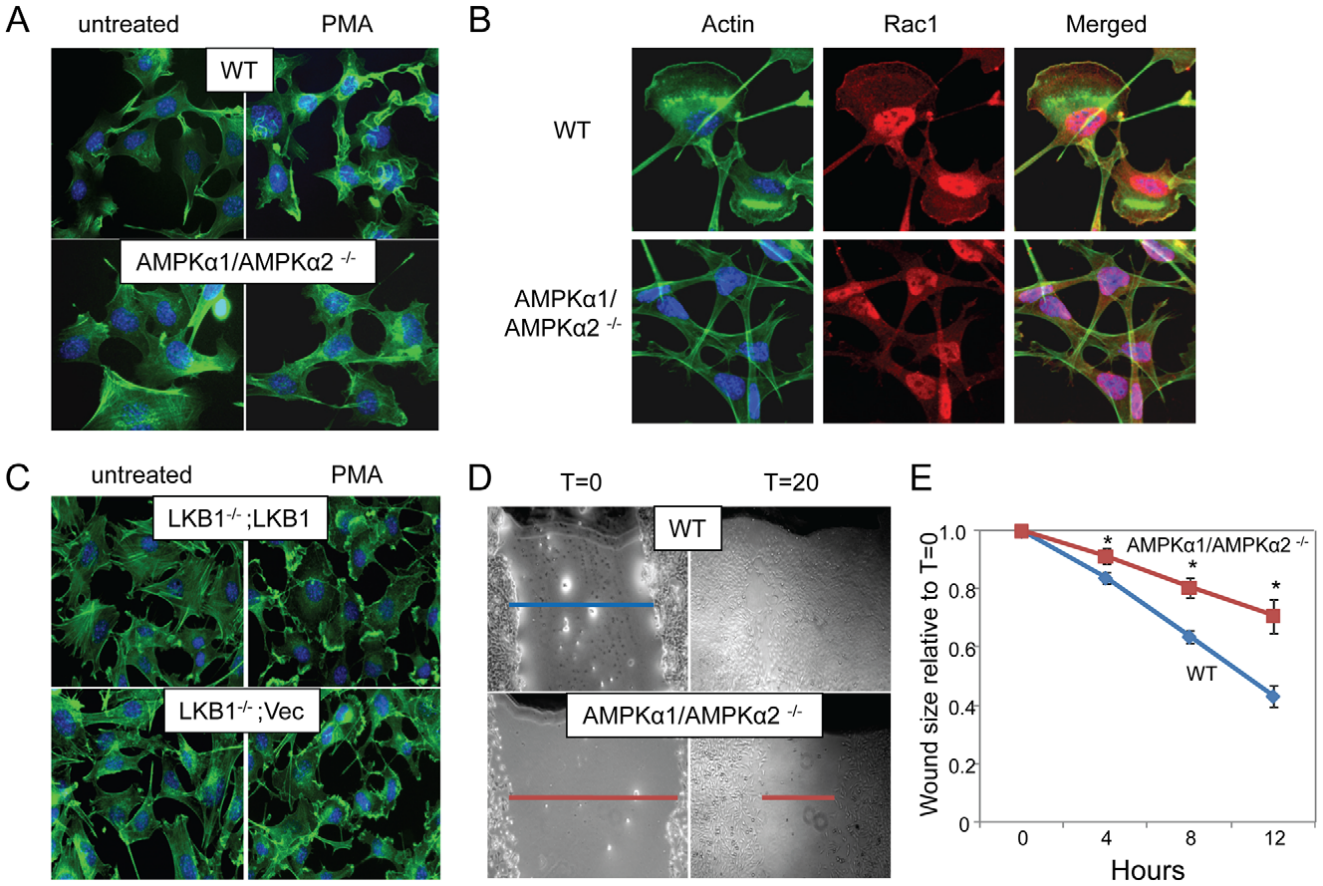
AMPK is required for actin-dependent membrane ruffling and wound healing independent of LKB1. **A**. Cells were treated with vehicle or PMA and stained with phalloidin (actin, green) and Hoescht 33342 (nuclei, blue). Arrows indicate lamellipodia. Representative images from triplicate experiments are shown. **B**. Cells were treated with PMA and stained with phalloidin (actin, green), anti-Rac1 (red) and Hoescht 33342 (nuclei, blue). Representative images from triplicate experiments are shown. **C**. MEFs null for LKB1 (LKB^−/−^; Vec) or rescued (LKB1^−/−^; LKB1) were treated with PMA or vehicle and stained as in **A**. Arrows indicate lamellipodia. Representative images from triplicate experiments are shown. **D**. A scratch was made in a confluent monolayer of wild type or AMPKα1/AMPKα2 ^−/−^ MEFs, and monitored over time for closure. Representative images from triplicate experiments are shown immediately after wounding (T = 0) and after 20 hours (T = 20). **E**. Reduction in wound width is quantified over time. Data are normalized to initial would width at T = 0, and presented as means of three independent experiments with four wounds per set; * indicates p<0.05 in each experiment. Moreover, the rate of wound healing is reduced in AMPK mutants by ANOVA (p<0.001).

One of the early steps in macropinocytosis involves extensive actin remodeling characterized by membrane ruffling and lamellipodia formation. We were interested in determining whether AMPK was required for this early step in the macropinocytic pathway, and whether the requirement for AMPK in macropinocytosis was vaccinia-dependent or if AMPK was required more generally for actin remodeling. Therefore, to test whether AMPK controlled actin-dependent remodeling independent of viral infection, we treated cells with phorbol myristic acid (PMA), which induces cells to undergo high levels of actin-mediated ruffling and lamellipodia formation; this is dependent on Rac1, a small Rho family GTPase that is also required for macropinocytosis and vaccinia infection [7] [35] [36] [37]. Dramatic lamellipodia formation, seen as thick bands of actin at the cell periphery, were observed in wild type MEFs stimulated with PMA, but abrogated in AMPK-deficient cells (Figure 7A, arrows). We also monitored PMA-induced ruffling using live cell imaging and observed a significant defect in the AMPK mutant MEFs (Videos S1, S2, Text S1).

In addition, we monitored the localization of Rac1 during PMA-induced lamellipodia formation in the wild type and AMPK deficient cells and found that both Rac1 re-localization and actin mobilization are defective in AMPK deficient cells (Figure 7B) suggesting that the defect is upstream of or parallel to Rac1 activation.

While our studies identified AMPK as a critical kinase required for vaccinia infection and actin dynamics, we found that LKB1 was dispensable for infection. This led us to test whether actin-dependent lamellipodia formation and ruffling was also LKB1-independent. We found that there was no defect in PMA-induced lamellipodia and ruffling in LKB1-deficient cells (Figure 7C, arrows). This was not unexpected since HeLa cells, a cell type routinely used for studies on actin dynamics, are mutant for LKB1, and can still undergo lamellipodia formation, and macropinocytosis [4] [38] [39] [40]. Therefore, we found that the actin remodeling activity of AMPK is independent of LKB1.

### AMPK promotes cellular motility

Since extensive actin remodeling and Rac1 membrane localization are required for lamellipodia formation, macropinocytosis, and vaccinia infection [7] [35] [36] [37], we tested whether AMPK was required for another Rac1-dependent actin-dependent process namely *in vitro* wound healing [41]. For these studies, we created wounds in a confluent monolayer of either wild type or AMPKα1/AMPKα2^−/−^ MEFs by scratching the surface, and monitored wound closure over time. Using this assay we found that there was a significant delay in the migration of AMPK-deficient MEFs into the wound compared to wild type cells (Figure 7D, E). While the wound was completely healed 24 hours after wounding in wild type cells, a sizable gap in the monolayer was still present in AMPK deficient cells, indicating a delay in wound healing and reduced motility. During this motile state, cells undergo a dramatic change in shape, with lamellipodia forming at the leading edge directing cell migration to close the wound. We observed the formation of lamellipodia in the WT MEFs at the edge of the wound while AMPK mutant MEFs did not polarize (Figure S13). Consistent with our observations that the role for AMPK in actin dynamics is LKB1-independent, we found that wound healing is unaffected by the loss of LKB1 (Figure S14).

## Discussion

Cell penetration is a critically important step in viral infection, and one that is generally driven by cellular factors and signaling pathways. First, viruses must attach to the cell surface and bind to the viral entry receptor, which often initiates signaling within the cell. Next, viruses must fuse or penetrate the cellular membrane either at the cell surface, or in many cases, within intracellular compartments by taking advantage of the endogenous endocytic machinery. Since there is an array of different endocytic mechanisms, there is great diversity in the strategies used by viruses for entry. Therefore, studying virus entry has increased our understanding not only of viral infection, but also of the normal cellular processes of endocytosis [6].

To further dissect the cellular signaling requirements of vaccinia entry, we developed an unbiased loss-of-function screening platform in *Drosophila* cells, and identified seven cellular factors required for vaccinia infection. Amongst the genes were all three subunits of AMPK, implicating the entire complex in vaccinia infection. Further studies showed that in addition to its role in *Drosophila*, AMPK is also required in murine and human cells for poxvirus infection at the stage of entry. Moreover, AMPK becomes rapidly activated upon infection with vaccinia, suggesting that virus-induced signals converge on this complex to facilitate entry.

Studies indicate that vaccinia virus can enter cells through multiple routes via as of yet unidentified receptor(s). Studies suggest that virus particles can fuse either at the plasma membrane or from within endosomal compartments, dependent on cell type and virus strain [42] [43] [44] [45] [46]. Importantly, a major endocytic pathway for vaccinia entry has recently been described as macropinocytosis [4] [47]. Our data as well as previous reports support the idea that vaccinia uses macropinocytosis for entry, but not exclusively. We and others have shown partial inhibition of vaccinia infection using a variety of drugs that are well-established inhibitors of macropinocytosis [4] [7]. However, in no case was vaccinia entry completely dependent upon macropinocytosis for infection, demonstrating that the virus can use alternative routes for entry. Nevertheless, macropinocytosis is required for efficient entry across broad cell types suggesting that inhibition of this pathway may attenuate infection sufficiently to allow for immune-mediated clearance of the infection.

The process of macropinocytosis drives non-specific uptake of extracellular fluid, large portions of the plasma membrane, as well as large particles. Macropinosomes, unlike coated vesicles, are morphologically heterogeneous, and can vary greatly in size from 0.2–10 µm in diameter, sufficient to accommodate the large size of vaccinia virus particles (∼0.3 µm) [5] [48]. Classic induction of macropinocytosis by growth factor receptor signaling stimulates ruffles, or sheet-like extensions of the plasma membrane, formed by assembly of actin filaments beneath the plasma membrane that form cups that contract and close to form macropinosomes [5]. This process is driven by signaling events initiated at the plasma membrane and are thought to involve a number of kinase families including phosphatidylinositol 5-kinases (PI5K), PI3K, PKC, serine/threonine kinases, and receptor tyrosine kinases. In addition, as many different inducers of macropinocytosis have been identified, there are likely multiple pathways that converge on the activation of macropinocytosis, adding to the complexity of this cell biological pathway [5]. While several specific kinases, such as PAK1, and LIM kinase have well described roles in macropinocytosis [49] [50], there is much that remains unclear. Here, we have found a role for an additional kinase, AMPK, in promoting vaccinia entry through its role in macropinocytosis. We have found that AMPK deficiency attenuates vaccinia infection, concomitant with reduced entry and fluid-phase uptake, supporting an important role for AMPK in vaccinia-induced macropinocytic entry.

The process of macropinocytosis involves several steps including extensive actin-mediated membrane ruffling, cup formation, and finally cup closure, which requires the fusion of plasma membranes to close off the macropinosome, followed by fission to separate the macropinosome from the plasma membrane [5]. We discovered that AMPK is required for the formation of lamellipodia and affects the recruitment of Rac1 to the cell periphery, suggesting that the role of AMPK in macropinocytosis lies in the initial rearrangement and reorganization of the actin cytoskeleton.

While we have shown that AMPK contributes to actin remodeling during vaccinia-induced macropinocytosis, we also found that AMPK plays a role in other virus-independent remodeling processes including cell migration. During this process, forward movement is driven by the extension of a leading edge protrusion or lamellipodium, followed by contraction at the rear. This protrusive force is generated by localized polymerization of actin mediated by Rac1 [51]. In addition to its role in controlling ruffling upstream of macropinocytosis, we found AMPK also has an essential role in cellular motility and wound healing, demonstrating a broad role in Rac1-dependent actin modulation. Previously, Rac1 has been implicated in nitric oxide production and glucose uptake downstream of AMPK [52] [53]. This role for AMPK and Rac1 in glucose uptake via the translocation of the major insulin-responsive glucose transporter GLUT-4 is quite interesting because this may provide a direct link between AMPK's role in energy homeostasis and the cytoskeleton [54] [55] [56].

While the best understood role of AMPK is its role in metabolism, recent evidence suggests this kinase also has a crucial role in regulating cell structure and polarity through engagement with the actin cytoskeleton. In *Drosophila*, loss of AMPK leads to defects in mitotic division and epithelial cell polarity accompanied by disruption of the actin cytoskeleton [29]. In some mammalian epithelial cell lines, AMPK activation leads to polarization characterized by the formation of an actin brush-border or tight junction assembly [29] [57] [58]. Additionally, AMPK activation can induce astrocyte stellation, and actin stress fiber disassembly [59]. Finally, studies using Compound C and AMPK activators linked AMPK to macropinocytic uptake of albumin in murine macrophages [60]. Taken together with our new data, this accumulating evidence suggests a broad and conserved role for AMPK in a variety of cellular processes that require actin cytoskeletal rearrangements.

The precise signaling events that lead to these AMPK-dependent cytoskeletal changes remain unclear. Several upstream kinases have been shown to activate AMPK under different stimuli. The best studied of these is the tumor suppressor LKB1 which activates AMPK in response to changes in cellular energy levels [27] [28]. Additionally, AMPK can be activated in response to changes in intracellular calcium levels by CaMKKβ, and further evidence suggests that TGFβ-activating kinase (TAK1) may serve as a third upstream activator [31] [32] [61]. Previous studies demonstrated that LKB1 is an important mediator of cell polarity at least in part through signaling to AMPK, and has been shown to drive actin brush border formation, and the translocation of apical and basal markers during the establishment of polarity [29] [30] [57] [58] [62]. We found that at least some AMPK-dependent cytoskeletal changes are independent of LKB1 and CaMKK including lamellipodia formation, macropinocytosis and wound healing. These different actin-dependent outcomes could be controlled by the unique downstream Rho GTPase family members that may become activated by AMPK depending on the stimulus and upstream kinase (such as Rac1, which is associated with lamellipodia formation and macropinocytosis) [10] [11]. Study of the Rho family GTPases activated by AMPK under different stimuli may resolve some of these issues.

In addition to the three subunits of AMPK discovered through screening the kinome, we identified four other genes that promote vaccinia infection: Pi3K68D, Fab1, Stam, and CG9311, all of which have human homologs. Pi3K68D, Fab1, and Stam are kinases while CG9311 is the only phosphatase identified in the screen. As kinases are druggable targets, and many known factors involved in macropinocytosis are kinases, we are particularly interested in the role of these kinases in vaccinia infection. Both Pi3K68D (PIK2C2A) and Fab1 (PIP5K3/PIKfyve) have roles in metabolism of phosphatidylinositol (PtdIns), an important component of membrane trafficking, cytoskeletal rearrangements, and macropinocytosis. In particular, Pi3K68D is a member of the class II family of PI3Ks that produce PtdIns(3)P downstream of growth factor stimulation, and can modulate the activity of Rho GTPases such as Rac1 and Cdc42. Class II PI3Ks have a critical role in lamellipodia formation and in cell migration, localizing to the leading edge of migrating cells [63] [64]. Interestingly, class I PI3K have also been implicated in vaccinia infection, during virus entry, and also later stages of infection [4] [65] [66]. Fab1, the PtdIns(3)P 5-kinase that converts PtdIns(3)P into PtdIns(3,5)P_2_, has been implicated in fluid-phase uptake, transport, and endosomal acidification [67] [68]. The third kinase, Stam (STAM, STAM2) is activated by cytokine and growth factor stimulation, and localizes to early endosomes, where it is involved in endosomal sorting [69] [70]. Since these kinases have roles either in trafficking to or from the plasma membrane, or in cytoskeletal rearrangements, and have been implicated in processes related to macropinocytosis, it is quite possible that they also play a direct role vaccinia entry. Perhaps Pi3K68D is involved in promoting cytoskeletal rearrangements that lead to macropinocytosis, while Fab1 and Stam could be involved sequentially in later entry steps leading to membrane fusion once a macropinosome has been internalized.

Rearrangements in the actin cytoskeleton are crucial not only for vaccinia infection, but also for many essential cellular processes including: cell division, establishment and maintenance of polarity, cellular motility, and uptake of extracellular fluids, each of which must be carefully regulated. While these various processes have different outcomes for the cell, they share several important signaling components, with AMPK as a central mediator. Further characterization of AMPK as well as these additional new factors is required to determine their precise role in vaccinia infection and whether they interact with AMPK, macropinocytosis, or other actin-dependent processes. How AMPK activation in response to different signals leads to these disparate changes in the actin cytoskeleton, and how these processes fit into the larger network of AMPK-dependent pathways will drive future studies. Lastly, the development of more selective AMPK inhibitors or other inhibitors of macropinocytosis may be useful against poxviruses, and other viruses that hijack this endocytic route for their entry mechanism.

## Materials and Methods

### Cells, antibodies, reagents, and viruses

*Drosophila* DL1 cells were grown and maintained at 25°C in Schneiders *Drosophila* media supplemented with 10% FBS (JRH) as described [71]. MEFs, BSC-1 and U2OS cells were maintained at 37°C in DMEM supplemented with 10% FBS (Sigma) and 10 mM Hepes. HeLa S3 suspension cells were maintained in MEM supplemented with 10% FBS and 0.05% Pluronic. BSC-1 cells were maintained in MEM supplemented with 10% cosmic calf serum (Hyclone). All media were additionally supplemented with 100 µg/ml penicillin/streptomycin and 2 mM L-glutamine. LKB1^−/−^ MEFs were complemented with MIGR (Vector) or FLAG-LKB1-MIGR (LKB1 cDNA) and maintained as above. Vaccinia strains vPRA13, vSC8, and vP30CP77, were grown in HeLa S3 suspension cells supplemented with 2.5% FBS, and tittered on BSC-1 cells as described [72] [73] [74] [75]. Cowpox Brighton Red and Vesicular Stomatitis virus (Indiana) were used. Antibodies were obtained from the following sources: anti-Bgal (Promega and Cappel), anti-E3L (gift from S. Isaacs) [76], anti-L1R (R180 gift from G. Cohen and R. Eisenberg), anti-Rac1 (Millipore),and anti-AMPK (Cell Signaling Technology). Fluorescently labeled secondary antibodies along with anti-sheep HRP were obtained from Jackson Immunochemicals or Invitrogen. All other HRP-conjugated antibodies were obtained from Amersham. AlexaFluor 488 and 594 phalloidin, FITC-conjugated dextran, and 594-conjugated Transferrin were purchased from Invitrogen. Compound C [20] and STO-609 [77] were obtained from Calbiochem. Additional chemicals were obtained from Sigma.

### RNAi and infections

A mini library of dsRNAs generated against *Drosophila* kinase and phosphatase genes was obtained from N. Perrimon, and aliquoted onto 384 well plates at 250 ng dsRNA/384 well [78]. Secondary amplicons and control dsRNA were designed using SnapDragon and DRSC resources (www.flyrnai.org), and generated as described [79]. For 384 well assays, 16,000 DL1 cells were seeded onto 250 ng dsRNA in 10 µl serum free media. One hour later 20 µl complete media was added, and cells were incubated in a humid chamber for 3 days. For other experiments, 2,000,000 cells were seeded onto 4 µg of dsRNA/6 well in 1 mL serum free media. One hour later 2 mL complete media was added. For viral infections, vaccinia was tittered on BSC-1 cells, and MOIs added to all cell types are based on pfu/ml measured on BSC-1 cells. Media was removed and virus was added in 2% serum medium and incubated at 25°C for *Drosophila* cells, and 37°C for mammalian cells. Viral innocula used was adjusted to achieve ∼10% infection of *Drosophila* cells in the primary screen, and ∼20% infection in secondary analysis. Level of infection of mammalian cells varied depending on the assay format. Cells were processed at the indicated time point post infection.

### Viral immunofluorescence

Cells were fixed and processed for immunofluorescence as previously described at 48 hours post infection for *Drosophila* cells and 8 hours post infection in mammalian cells [12]. Briefly, cells were fixed in 4% formaldehyde/phosphate buffered saline (PBS), washed twice in PBS/0.1% TritonX-100 (PBST), and blocked in 2% BSA/PBST. Anti-E3L and anti-B-gal primary antibodies were diluted in block, added to cells, and incubated overnight at 4°C. Cells were washed three times in PBST, and incubated in secondary antibody for one hour at room temperature. Cells were counterstained with Hoescht33342 (Sigma). Plates were imaged at 20X for *Drosophila* cells and 10X for mammalian cells, capturing three images per well per wavelength using an automated microscope (ImageXpress Micro), and quantification was performed using MetaXpress image analysis software. Significance was determined using a Student T-test.

### Screen analysis

Image analysis was used to generate metrics from the captured images including the number of nuclei and the number of infected cells per site. The percent infection was calculated for each site, log-transformed, and the interquartile range (IQR) was used to calculate a robust Z score for each site using the following equation: log_10_ [(%infection-median)/(IQR*0.74)] [80]. Candidates were identified as positive if the average robust Z score of all sites in a well was <−2 in two independent replicates.

### Immunoblotting, Northern blotting, and RT-PCR

For protein analysis, MEFs were prechilled to 16°C for 10 minutes and then treated with vaccinia (MOI 20) for 1 hour at 16°C to synchronize infection. Cells were incubated at 37°C for 10 or 30 minutes or treated with 10 µM 2DG for 30 minutes. Cells were then washed briefly in cold PBS and lysed in NP40 lysis buffer supplemented with protease (Boehringer) and phosphatase (Sigma) inhibitor cocktails. Samples were separated by SDS-PAGE and blotted as described [81]. HRP-conjugated secondary antibodies and Western Lightening Chemiluminescence Reagent were used for visualization.

For RNA analysis, cells were lysed in Trizol buffer, and RNA was purified and blotted as previously described with the indicated probes [12]. RT-PCR was performed using M-MLV reverse transcriptase on random primed total RNA (Invitrogen). One µL of the cDNA or a 1∶10 dilution was used for PCR amplification.

### Plaque assays

Viruses were plaqued on MEF or BSC-1 cells as indicated. Confluent monolayers were treated with serial dilutions of virus for two hours, after which the cells were overlayed with agarose followed by crystal violet staining. Plaque number was determined manually, and plaque diameter was measured using MetaXpress software and used to calculate areas.

### Vaccinia entry assay

MEFs plated on cover slips were chilled to 16°C for 10 minutes and then treated with vaccinia (MOI 100) at 16°C for 1 hour. Unbound virus was removed, and cells were incubated at 37°C for 1 hour, washed three times in cold PBS, and fixed. Cells were washed in ammonium chloride (50 mM) and PBST and were stained with anti-L1R and then washed and incubated with secondary antibody, Hoescht 33342, and phalloidin 488. Cover slips were mounted and imaged using a 63X objective with a Leica DMI 4000 B fluorescent microscope. Images were taken as 0.2 um Z-stacks that were deconvolved using AutoQuant X2 software using Adaptive PSF with 20 iterations. Images are displayed as max projections. To quantify, images were randomized and blindly quantified for virus entry (n>30 for each condition).

### Fluid-phase dextran uptake assay

MEFs grown on glass cover slips were chilled to 16°C for 10 minutes and then treated with vaccinia (MOI 200) at 16°C for 1 hour. Unbound virus was removed, and FITC-dextran (70 kD, lysine fixable) was added at 0.5 mg/ml. Cells were incubated at 37°C for 20 minutes, washed twice in PBS, and once in pH 5.5 buffer (0.1 M sodium acetate, 0.05 M NaCl) for 5 minutes. Cells were fixed and stained with Hoescht 33342 and phalloidin 594. Cover slips were mounted and imaged using a 63X objective with a Leica DMI 4000 B fluorescent microscope. Images were randomized and blindly quantified for the percentage of cells undergoing macropinocytosis as defined by >20 punctae per cell. U2OS cells grown on glass cover slips were pretreated with 10 µM Compound C or vehicle for 1 hour and then assayed as above.

### Actin ruffling assay

Cells were grown on glass cover slips and treated with vehicle or 1 µM PMA for 3 hours. For Rac1 localization experiments, cells were blocked in 8% BSA/PBST for 1 hour. Anti-Rac1 (Millipore) was added in 1% BSA/PBST overnight at 4°C. Cells were washed 3 times in PBST, and secondary antibodies were added for 1 hour at room temperature. For all experiments, cells were stained with Hoescht 33342 and phalloidin 488. Cover slips were mounted and imaged using a 63X objective with a Leica DMI 4000 B fluorescent microscope.

### Transferrin uptake assay

Cells grown on glass cover slips were chilled to 16°C for 10 minutes and then treated with vaccinia (MOI 100) at 16°C for 1 hour. Unbound virus was removed, and 594-transferrin was added at 20 µg/ml. Cells were incubated at 37°C for 20 minutes, washed twice in PBS, and once in 0.1 M sodium acetate, 0.05 M NaCl, pH 5.5 buffer for 5 minutes. Cells were fixed and stained with Hoescht 33342 and phalloidin 488. Cover slips were mounted and imaged using a 63X objective with a Leica DMI 4000 B fluorescent microscope.

### Wound healing assay

Cells were grown to 100% confluence overnight, then scratched with a pipet tip to wound. Several marks were made along the length of the wound, and were imaged over time, using these marks as guides. Images were analyzed for wound length at the same position over time using MetaXpress software.

## Supporting Information

Text S1 Supplemental experimental procedures.
(0.09 MB PDF)

Figure S1 Vaccinia infection in *Drosophila* cells. **A.** *Drosophila* DL1 cells were infected with vaccinia virus expressing B-gal driven by an early/late promoter (p7.5) for indicated time, and stained for X-gal production. A representative of 2 experiments is shown. **B.** Titration of vaccinia infection in *Drosophila* cells seeded in 384 well plates. Cells were fixed and processed 48 hpi and stained for early B-gal expression (green) and nuclei (blue). **C.** Quantification of B. Percent infection is the average of 6 wells, with 3 images per well in duplicate experiments. Bars represent average percent infection for each experiment.
(2.10 MB TIF)

Figure S2 Inhibitors of macropinocytosis inhibit vaccinia infection in mammalian and *Drosophila* cells. **A.** Human U2OS cells were pretreated with: Latrunculin A (Lat A, 5 µM), Wortmannin (Wort, 5 µM), Rottlerin (10 µM), or EIPA (12.5 µM) for 1 hour, challenged with vaccinia (MOI = 10) for 8 hours, and quantified for percent infection. **B.** *Drosophila* DL1 cells were treated with: Latrunculin A (Lat A, 5 µM), Wortmannin (Wort, 5 µM), and Rottlerin (5 µM), or EIPA (50 µM) for 1 hour and challenged with vaccinia (MOI = 20) for 24 hours. Cells were fixed and processed for immunofluorescence using E3L expression as a marker for infection, and Hoescht 33342 to visualize nuclei. Mean percent infection + SD in triplicate experiments is shown; * indicates p<0.05 compared to control in three independent experiments.
(0.13 MB TIF)

Figure S3 Validation of eight candidates that promote vaccinia infection identified in RNAi screen of *Drosophila* kinases and phosphatases. Independent dsRNA targeting different sequences of each candidate gene were tested, and percent infection was determined by immunofluorescence measuring B-gal expressing cells. Luciferase was used as a nontargeting negative control. B-gal and Rab5 were added as positive controls for decreased infection. A representative of duplicate experiments is shown. Error bars represent standard deviation of 12 different wells with 3 images taken per well. * indicate p-value of <0.001 in both experiments.
(0.16 MB TIF)

Figure S4 dsRNA against AMPKα or LKB1 leads to depletion of the cognate mRNA in *Drosophila* cells. RNAi was performed against luciferase (luc) or AMPKα (**A**) or LKB1 (**B**) in *Drosophila* cells. RNA was collected from lysates and RT-PCR was performed to measure mRNA levels. A 1∶10 dilution of control cDNA (luc) was included to demonstrate that the depletion was greater than 10-fold. Clathrin heavy chain (chc) was used as a loading control.
(1.75 MB TIF)

Figure S5 AMPKα1/AMPKα2 ^−/−^ MEFs do not express AMPKα. Wild type or AMPKα1/AMPKα2 ^−/−^ MEF protein lysates were collected and probed by immunoblot for total-AMPKα and tubulin.
(0.26 MB TIF)

Figure S6 AMPK promotes efficient cowpox virus infection. Plaque assays were performed on wild type or AMPKα1/AMPKα2 ^−/−^ MEFs and quantified in duplicate experiments. Error bars show the individual values; * p<0.05 in each replicate.
(0.26 MB TIF)

Figure S7 AMPK is not required for Vesicular Stomatitis Virus infection. Plaque assays were performed on wild type or AMPKα1/AMPKα2 ^−/−^ MEFs. There was no decrease in plaque number observed in the mutant cells. A representative experiment of three is shown.
(1.01 MB TIF)

Figure S8 AMPK promotes early vaccinia infection in mammalian cells. **A.** Lack of AMPKα leads to decreased vaccinia infectivity in MEFs. Wild type or AMPKα1/AMPKα2 ^−/−^ MEFs were infected with the indicated MOI for 8 hours and processed for immunofluorescence. Data is displayed as average percentage of infected cells for a representative experiment. **B.** Loss of AMPKα leads to a decrease in viral mRNA production in AMPKα1/AMPKα2 ^−/−^ MEFs. Northern blot of viral mRNA levels in WT or AMPKα1/AMPKα2 ^−/−^ MEFs at indicated times post infection (MOI = 10). Blots were probed for virally encoded E3L or a ribosomal RNA loading control. **C.** Wild type or AMPKα1/AMPKα2 ^−/−^ cells were infected (MOI = 10) for the indicated times and probed for E3L by immunoblot.
(0.51 MB TIF)

Figure S9 siRNA targeting AMPK inhibits vaccinia infection in mammalian cells. **A.** U2OS cells were treated with non-targeting siRNA (siCON) or siRNA targeting AMPKα1 or AMPKα2 and infected with vaccinia virus (MOI 10), and stained for E3L expression after 8 hours. **B.** Quantification of percent infection from A. The average of duplicate experiments; error bars represent mean of the percent infection for each experiment. **C.** Western blot probing total AMPKα after siRNA treatment.
(1.69 MB TIF)

Figure S10 LKB1 cDNA rescues the LKB1 null cells. LKB1 ^−/−^ MEFs were complemented with a vector control (Vec) or FLAG-LKB1 (LKB1) cDNA and were mock treated, or treated with 2-deoxyglucose (2DG) which leads to LKB1-dependent AMPK phosphorylation for 30 min. Protein lysates were collected and probed by immunoblot for FLAG, phospho- or total-AMPKα expression.
(0.72 MB TIF)

Figure S11 Vaccinia produced in AMPK deficient cells is infectious. **A.** AMPK is not required for late vaccina protein expression. WT and AMPKα1/AMPKα2 ^−/−^ MEFs infected with vaccinia for 8 hours were stained for early (E3L, green) and late (L1R, red) vaccinia protein expression. **B.** Infectious virus is produced in AMPK deficient cells. Vaccinia grown for 12 hours in WT and AMPKα1/AMPKα2 ^−/−^ MEFs was titered in BSC-1 cells. Rifampicin (Rif) was added as a control for detecting incoming virus. The relative pfu/ml in BSC-1 cells was graphed as the mean + standard deviation of triplicate experiments. The decrease in virus produced was similar to decrease in virus entry.
(1.53 MB TIF)

Figure S12 AMPK deficient cells undergo efficient receptor-mediated endocytosis. Transferrin uptake assays were performed in the presence or absence of virus. Wild type and AMPKα1/AMPKα2 ^−/−^ MEFs were either infected or mock-infected and treated with 594-transferrin (red), processed for microscopy, and stained tovisualize actin (phalloidin (green)) and nuclei (Hoescht 33342 (blue)). Representative images from triplicate experiments are shown.
(3.74 MB TIF)

Figure S13 AMPK deficient cells are defective in lamellipodia formation during wound healing. A scratch was made in a confluent monolayer of wild type or AMPKα1/AMPKα2 ^−/−^ MEFs, and monitored over time. Images were taken using a 20X and 63X objective immediately after wounding (T = 0), and again after 3 and 6 hours to determine the morphology of the cells at the wound front. Polarized cells with lamellipodia are visible at the wound front of wild type MEFs (arrows). Representative images from triplicate experiments are shown.
(3.43 MB TIF)

Figure S14 Cellular motility is LKB1-independent. Scratches were introduced into a confluent monolayer of LKB1 ^−/−^, Vec or LKB1 ^−/−^; LKB1 cDNA MEFs, and monitored over time for closure. Representative images from triplicate experiments are shown immediately after wounding (T = 0) and after 12 or 24 hours. The reduction in wound width is quantified over time. Data are normalized to initial would width at T = 0, and presented as means of three independent experiments with four wounds per set.
(3.01 MB TIF)

Table S1 Primary screen data.
(0.02 MB TXT)

Video S1 Wild type MEFs were treated with 1 uM PMA and imaged live at 10 second intervals for 30 minutes using DIC microscopy. Extensive ruffling was observed at the cell periphery.
(12.77 MB MOV)

Video S2 AMPKα1/AMPKα2 ^−/−^ MEFs were treated with 1 uM PMA and imaged live at 10 second intervals for 30 minutes using DIC microscopy. Cells remained quiescent and no ruffling was observed.
(9.96 MB MOV)
