# Supplementary material for: A Kinome RNAi Screen Identified AMPK as Promoting Poxvirus Entry through the Control of Actin Dynamics
Source: PLoS Pathog. 2010 Jun 17;6(6):e1000954. doi: 10.1371/journal.ppat.1000954 (PMC2887478; doi:10.1371/journal.ppat.1000954)
Supplement: Text S1 — Supplemental experimental procedures. (0.09 MB PDF) [file ppat.1000954.s001.pdf]

Supporting Online Material:

Supplemental Experimental Procedures

Transferrin uptake assay

Cells grown on glass coverslips were chilled to 16ºC for 10 minutes and then treated with vaccinia (MOI=100) at 16º C for 1 hour. Unbound virus was removed, and 594-transferrin was added at 20 ug/ml. Cells were incubated at 37º C for 20 minutes, washed twice in PBS, and once in pH 5.5 buffer (0.1 M sodium acetate, 0.05 M NaCl) for 5 minutes. Cells were fixed and stained with Hoescht 33342 and phalloidin 488. Coverslips were mounted and imaged using a 63X objective with a Leica DMI 4000 B fluorescent microscope.

siRNA

siRNAs were obtained from ABI. U2OS cells were reverse transefected with HiPerfect and incubated for 3 days followed by infection at MOI=10 for 8 hours, fixed, and processed for immunofluorescence.

Live Cell Imaging

Live cell imaging was performed on MEF cells grown on collagen-coated glass bottom 35mm dishes (Wilco Imaging). Dishes were transferred to a heated and CO_2 controlled DH-35i micro-incubation system (Warner Instruments) positioned over an Olympus IX81 equipped with a Hamamatsu Orca-R2 CCD camera and Sutter Lambda 10-3 High Speed filter wheel system. Differential interference contrast (DIC) images

were captured using an Olympus PL APO 60x/1.4 objective every 10 seconds prior to and following the addition of PMA (1μM) (Calbiochem) for a total of 30 min.

Supplementary Figure Legends

Figure S1: Vaccinia infection in *Drosophila* cells. A. *Drosophila* DL1 cells were infected with vaccinia virus expressing B-gal driven by an early/late promoter (p7.5) for indicated time, and stained for X-gal production. A representative of 2 experiments is shown. **B.** Titration of vaccinia infection in *Drosophila* cells seeded in 384 well plates. Cells were fixed and processed 48 hpi and stained for early B-gal expression (green) and nuclei (blue). **C.** Quantification of B. Percent infection is the average of 6 wells, with 3 images per well in duplicate experiments. Bars represent average percent infection for each experiment.

Figure S2: Inhibitors of macropinocytosis inhibit vaccinia infection in mammalian and *Drosophila* cells. A. Human U2OS cells were pretreated with: Latrunculin A (Lat A, 5μM), Wortmannin (Wort, 5μM), Rottlerin (10μM), or EIPA (12.5μM) for 1 hour, challenged with vaccinia (MOI=10) for 8 hours, and quantified for percent infection. **B**. *Drosophila* DL1 cells were treated with: Latrunculin A (Lat A, 5μM), Wortmannin (Wort, 5μM), and Rottlerin (5μM), or EIPA (50μM) for 1 hour and challenged with vaccinia (MOI=20) for 24 hours. Cells were fixed and processed for immunofluorescence using E3L expression as a marker for infection, and Hoescht 33342 to visualize nuclei. Mean percent infection + SD in triplicate experiments is shown; * indicates $p<0.05$ compared to control in three independent experiments.

Figure S3: Validation of eight candidates that promote vaccinia infection identified in RNAi screen of *Drosophila* kinases and phosphatases. Independent dsRNA targeting different sequences of each candidate gene were tested, and percent infection was determined by immunofluorescence measuring B-gal expressing cells. Luciferase was used as a nontargeting negative control. B-gal and Rab5 were added as positive controls for decreased infection. A representative of duplicate experiments is shown. Error bars represent standard deviation of 12 different wells with 3 images taken per well. * indicate p-value of <0.001 in both experiments.

Figure S4: dsRNA against AMPKα or LKB1 leads to depletion of the cognate mRNA in *Drosophila* cells. RNAi was performed against luciferase (luc) or AMPKα (**A**) or LKB1 (**B**) in *Drosophila* cells. RNA was collected from lysates and RT-PCR was performed to measure mRNA levels. A 1:10 dilution of control cDNA (luc) was included to demonstrate that the depletion was greater than 10-fold. Clathrin heavy chain (chc) was used as a loading control.

Figure S5: **AMPKα1/AMPKα2 $^{-/-}$ MEFs do not express AMPKα.** Wild type or AMPKα1/AMPKα2 $^{-/-}$ MEF protein lysates were collected and probed by immunoblot for total-AMPKα and tubulin.

Figure S6: AMPK promotes efficient cowpox virus infection. Plaque assays were performed on wild type or AMPKα1/AMPKα2 $^{-/-}$ MEFs and quantified in duplicate experiments. Error bars show the individual values; * p<0.05 in each replicate.

Figure S7: AMPK is not required for Vesicular Stomatitis Virus infection. Plaque assays were performed on wild type or AMPKα1/AMPKα2 $^{-/-}$ MEFs. There was no decrease in plaque number observed in the mutant cells. A representative experiment of three is shown.

Figure S8: AMPK promotes early vaccinia infection in mammalian cells. A. Lack of AMPKα leads to decreased vaccinia infectivity in MEFs. Wild type or AMPKα1/AMPKα2 $^{-/-}$ MEFs were infected with the indicated MOI for 8 hours and processed for immunofluorescence. Data is displayed as average percentage of infected cells for a representative experiment. **B**. Loss of AMPKα leads to a decrease in viral mRNA production in AMPKα1/AMPKα2 $^{-/-}$ MEFs. Northern blot of viral mRNA levels in WT or AMPKα1/AMPKα2 $^{-/-}$ MEFs at indicated times post infection (MOI=10). Blots were probed for virally encoded E3L or a ribosomal RNA loading control. **C**. Wild type or AMPKα1/AMPKα2 $^{-/-}$ cells were infected (MOI=10) for the indicated times and probed for E3L by immunoblot.

Figure S9: siRNA targeting AMPK inhibits vaccinia infection in mammalian cells. A. U2OS cells were treated with non-targeting siRNA (siCON) or siRNA targeting AMPKα1 or AMPKα2 and infected with vaccinia virus (MOI 10), and stained for E3L

expression after 8 hours. **B.** Quantification of percent infection from A. The average of duplicate experiments; error bars represent mean of the percent infection for each experiment. **C.** Western blot probing total AMPKα after siRNA treatment.

Figure S10: **LKB1 cDNA rescues the LKB1 null cells.** LKB1 $^{-/-}$ MEFs were complemented with a vector control (Vec) or FLAG-LKB1 (LKB1) cDNA and were mock treated, or treated with 2-deoxyglucose (2DG) which leads to LKB1-dependent AMPK phosphorylation for 30 min. Protein lysates were collected and probed by immunoblot for FLAG, phospho- or total-AMPKα expression.

Figure S11: Vaccinia produced in AMPK deficient cells is infectious. A. AMPK is not required for late vaccina protein expression. WT and AMPKα1/AMPKα2 $^{-/-}$ MEFs infected with vaccinia for 8 hours were stained for early (E3L, green) and late (L1R, red) vaccinia protein expression. **B.** Infectious virus is produced in AMPK deficient cells. Vaccinia grown for 12 hours in WT and AMPKα1/AMPKα2 $^{-/-}$ MEFs was titered in BSC-1 cells. Rifampicin (Rif) was added as a control for detecting incoming virus. The relative pfu/ml in BSC-1 cells was graphed as the mean + standard deviation of triplicate experiments. The decrease in virus produced was similar to decrease in virus entry.

Figure S12: **AMPK deficient cells undergo efficient receptor-mediated endocytosis.** Transferrin uptake assays were performed in the presence or absence of virus. Wild type and AMPKα1/AMPKα2 $^{-/-}$ MEFs were either infected or mock-infected and treated with 594-transferrin (red), processed for microscopy, and stained to

visualize actin (phalloidin (green)) and nuclei (Hoescht 33342 (blue)). Representative images from triplicate experiments are shown.

Figure S13: **AMPK deficient cells are defective in lamellipodia formation during wound healing.** A scratch was made in a confluent monolayer of wild type or AMPKα1/AMPKα2 $^{-/-}$ MEFs, and monitored over time. Images were taken using a 20X and 63X objective immediately after wounding (T=0), and again after 3 and 6 hours to determine the morphology of the cells at the wound front. Polarized cells with lamellipodia are visible at the wound front of wild type MEFs (arrows). Representative images from triplicate experiments are shown.

Figure S14: **Cellular motility is LKB1-independent.** Scratches were introduced into a confluent monolayer of LKB1 $^{-/-}$, Vec or LKB1 $^{-/-}$; LKB1 cDNA MEFs, and monitored over time for closure. Representative images from triplicate experiments are shown immediately after wounding (T=0) and after 12 or 24 hours. The reduction in wound width is quantified over time. Data are normalized to initial would width at T=0, and presented as means of three independent experiments with four wounds per set.

Supplementary Movie 1: Wild type MEFs were treated with 1uM PMA and imaged live at 10 second intervals for 30 minutes using DIC microscopy. Extensive ruffling was observed at the cell periphery.

Supplementary Movie 2: AMPKα1/AMPKα2 $^{-/-}$ MEFs were treated with 1uM PMA and imaged live at 10 second intervals for 30 minutes using DIC microscopy. Cells remained quiescent and no ruffling was observed.
