# Supplementary figures and images for: A Kinome RNAi Screen Identified AMPK as Promoting Poxvirus Entry through the Control of Actin Dynamics
Source: PLoS Pathog. 2010 Jun 17;6(6):e1000954. doi: 10.1371/journal.ppat.1000954 (PMC2887478; doi:10.1371/journal.ppat.1000954)

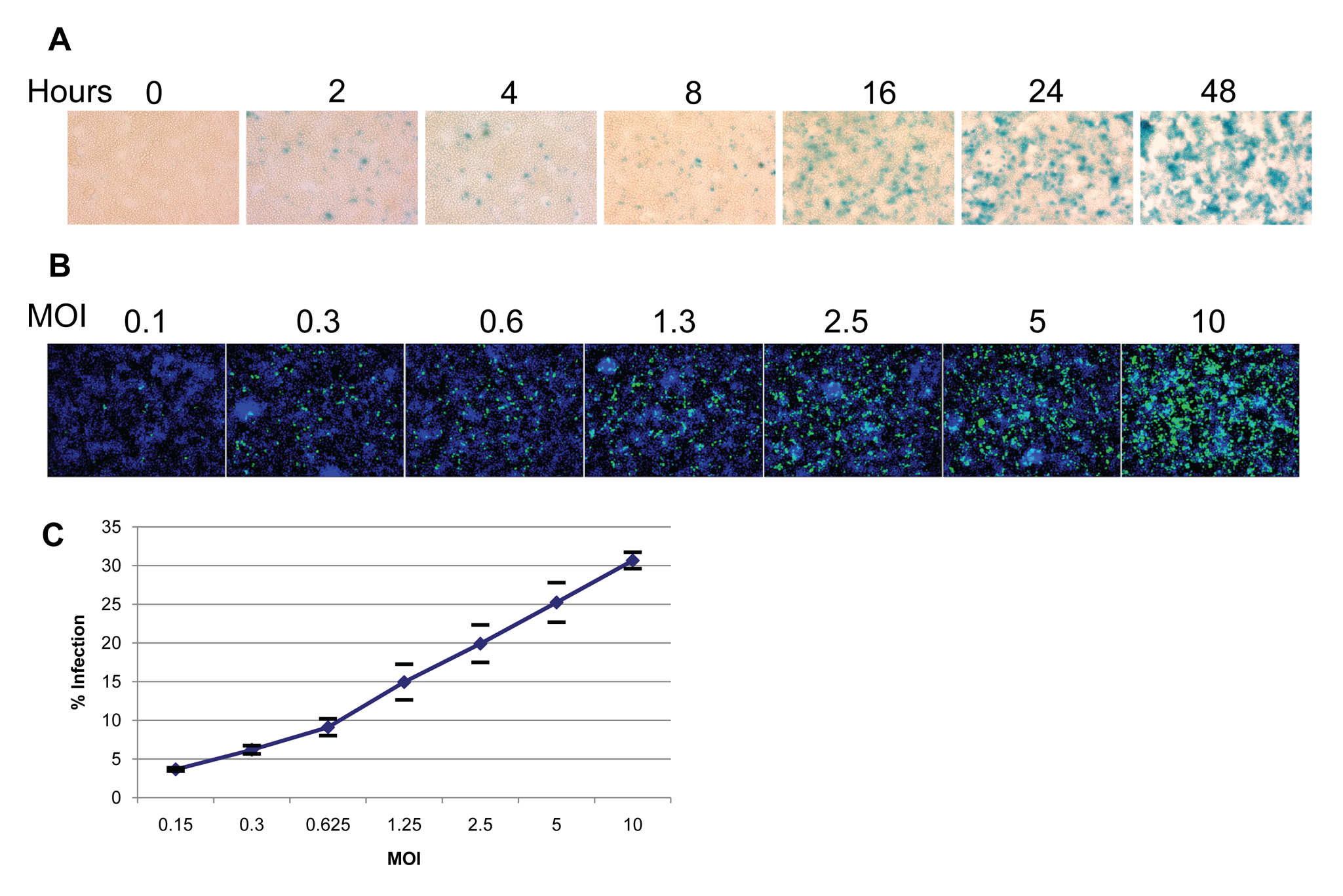

Supplement: Figure S1 — Vaccinia infection in Drosophila cells. A. Drosophila DL1 cells were infected with vaccinia virus expressing B-gal driven by an early/late promoter (p7.5) for indicated time, and stained for X-gal production. A representative of 2 experiments is shown. B. Titration of vaccinia infection in Drosophila cells seeded in 384 well plates. Cells were fixed and processed 48 hpi and stained for early B-gal expression (green) and nuclei (blue). C. Quantification of B. Percent infection is the average of 6 wells, with 3 images per well in duplicate experiments. Bars represent average percent infection for each experiment. (2.10 MB TIF) [file ppat.1000954.s002.tif]

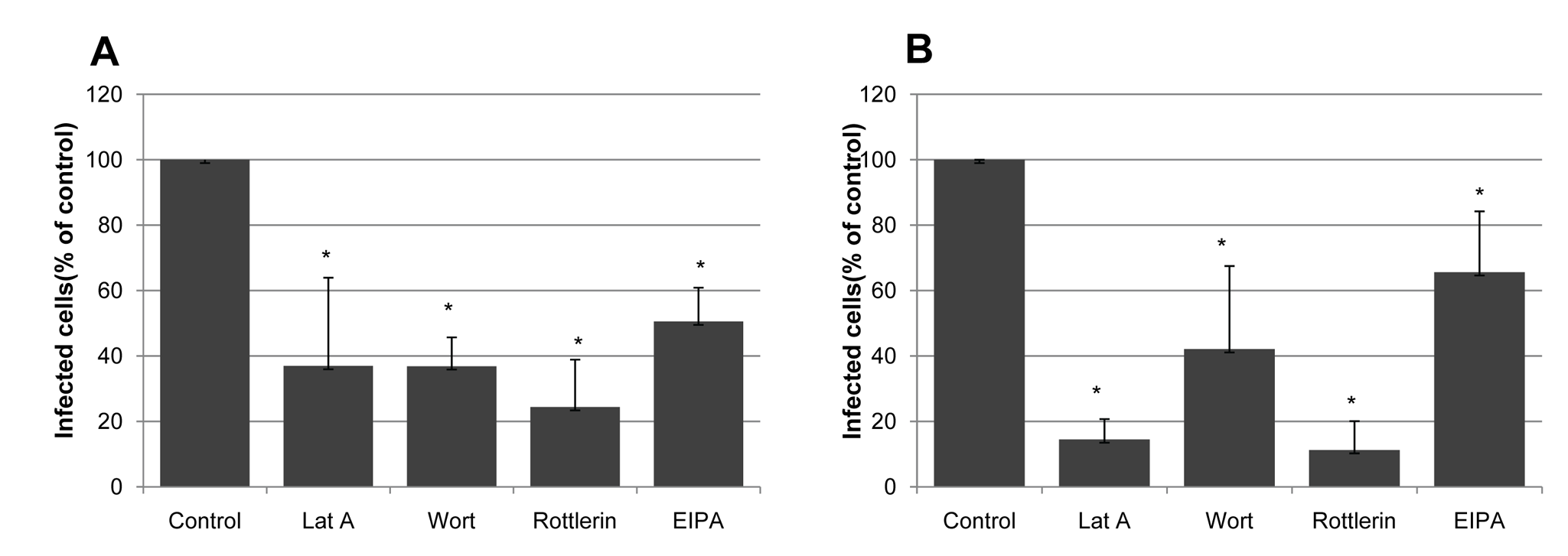

Supplement: Figure S2 — Inhibitors of macropinocytosis inhibit vaccinia infection in mammalian and Drosophila cells. A. Human U2OS cells were pretreated with: Latrunculin A (Lat A, 5 µM), Wortmannin (Wort, 5 µM), Rottlerin (10 µM), or EIPA (12.5 µM) for 1 hour, challenged with vaccinia (MOI = 10) for 8 hours, and quantified for percent infection. B. Drosophila DL1 cells were treated with: Latrunculin A (Lat A, 5 µM), Wortmannin (Wort, 5 µM), and Rottlerin (5 µM), or EIPA (50 µM) for 1 hour and challenged with vaccinia (MOI = 20) for 24 hours. Cells were fixed and processed for immunofluorescence using E3L expression as a marker for infection, and Hoescht 33342 to visualize nuclei. Mean percent infection + SD in triplicate experiments is shown; * indicates p<0.05 compared to control in three independent experiments. (0.13 MB TIF) [file ppat.1000954.s003.tif]

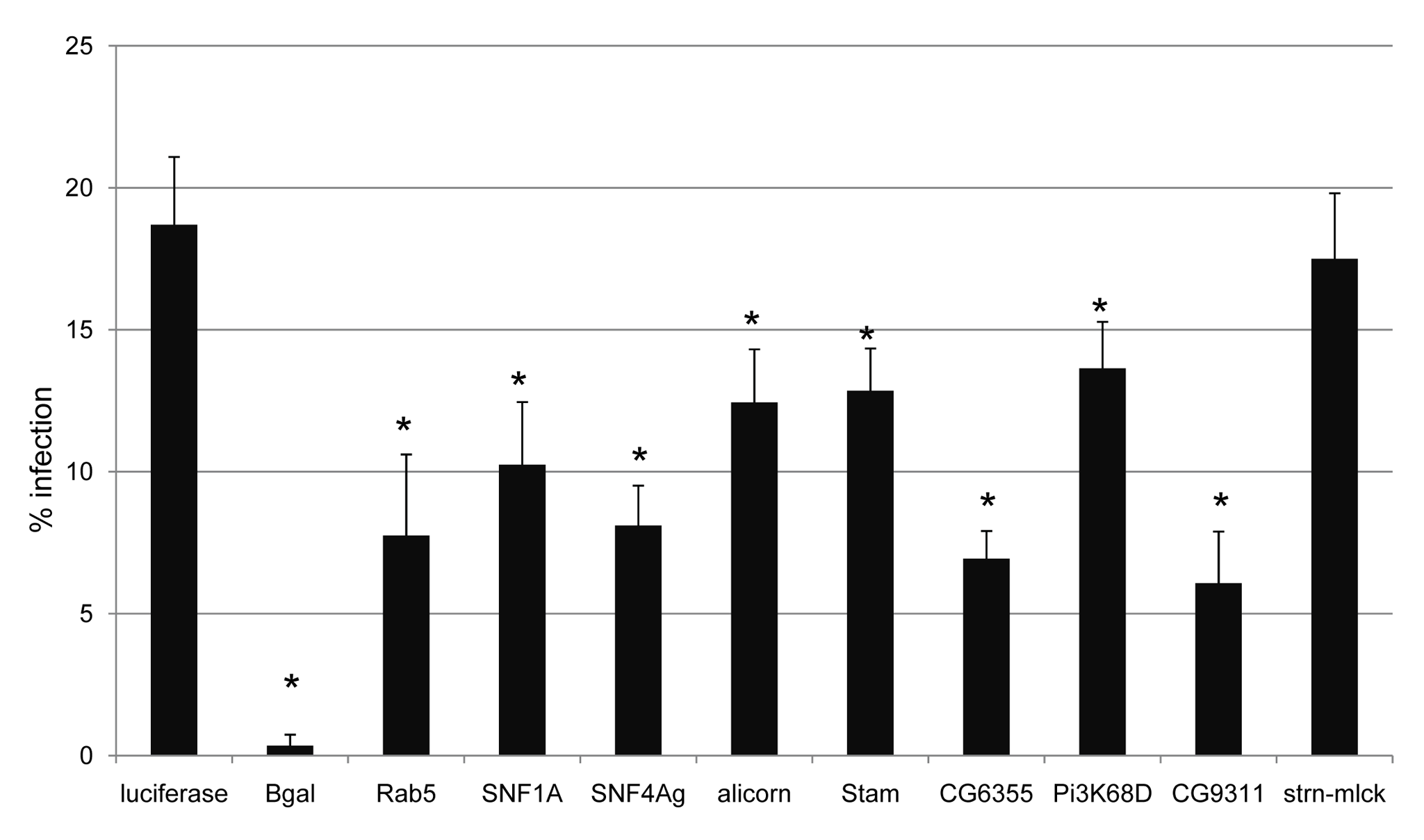

Supplement: Figure S3 — Validation of eight candidates that promote vaccinia infection identified in RNAi screen of Drosophila kinases and phosphatases. Independent dsRNA targeting different sequences of each candidate gene were tested, and percent infection was determined by immunofluorescence measuring B-gal expressing cells. Luciferase was used as a nontargeting negative control. B-gal and Rab5 were added as positive controls for decreased infection. A representative of duplicate experiments is shown. Error bars represent standard deviation of 12 different wells with 3 images taken per well. * indicate p-value of <0.001 in both experiments. (0.16 MB TIF) [file ppat.1000954.s004.tif]

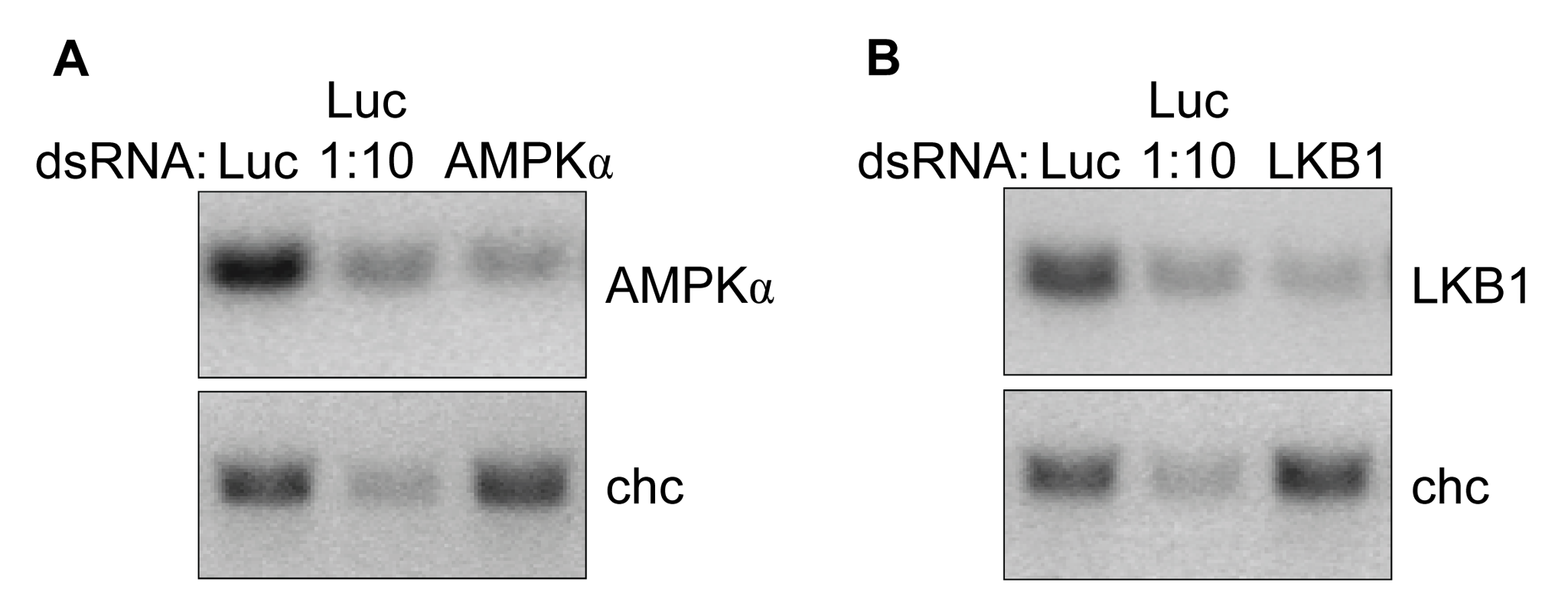

Supplement: Figure S4 — dsRNA against AMPKα or LKB1 leads to depletion of the cognate mRNA in Drosophila cells. RNAi was performed against luciferase (luc) or AMPKα (A) or LKB1 (B) in Drosophila cells. RNA was collected from lysates and RT-PCR was performed to measure mRNA levels. A 1∶10 dilution of control cDNA (luc) was included to demonstrate that the depletion was greater than 10-fold. Clathrin heavy chain (chc) was used as a loading control. (1.75 MB TIF) [file ppat.1000954.s005.tif]

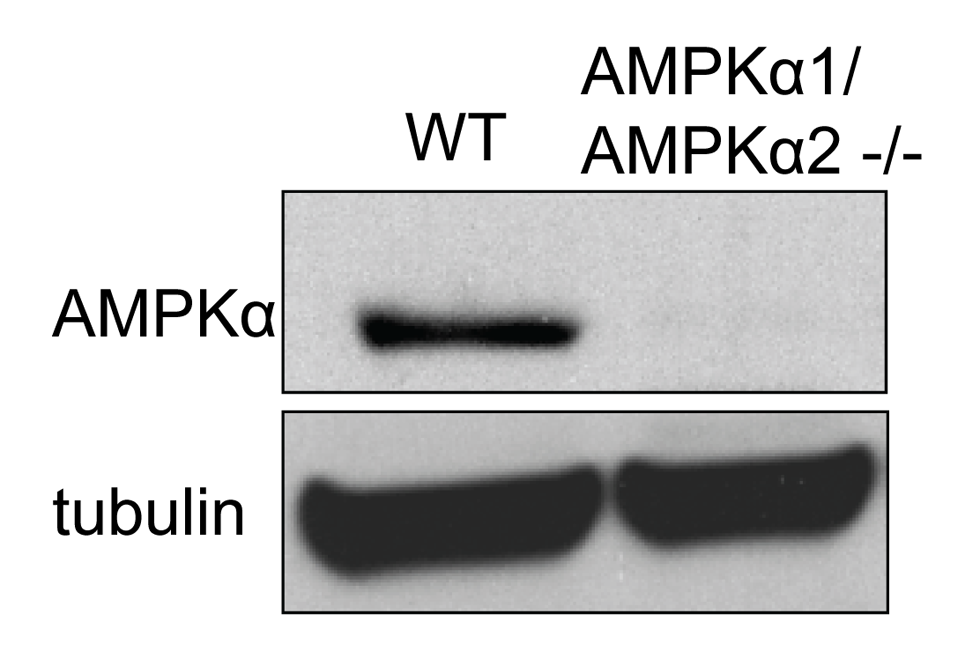

Supplement: Figure S5 — AMPKα1/AMPKα2 −/− MEFs do not express AMPKα. Wild type or AMPKα1/AMPKα2 −/− MEF protein lysates were collected and probed by immunoblot for total-AMPKα and tubulin. (0.26 MB TIF) [file ppat.1000954.s006.tif]

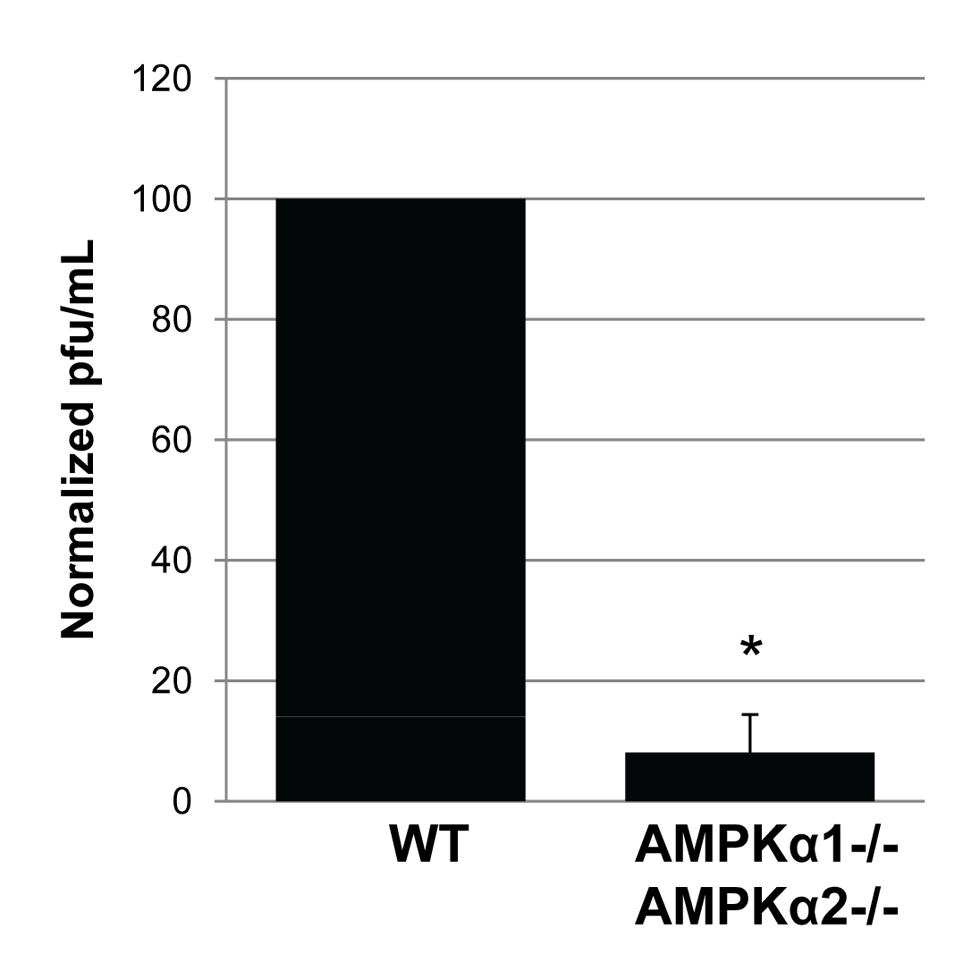

Supplement: Figure S6 — AMPK promotes efficient cowpox virus infection. Plaque assays were performed on wild type or AMPKα1/AMPKα2 −/− MEFs and quantified in duplicate experiments. Error bars show the individual values; * p<0.05 in each replicate. (0.26 MB TIF) [file ppat.1000954.s007.tif]

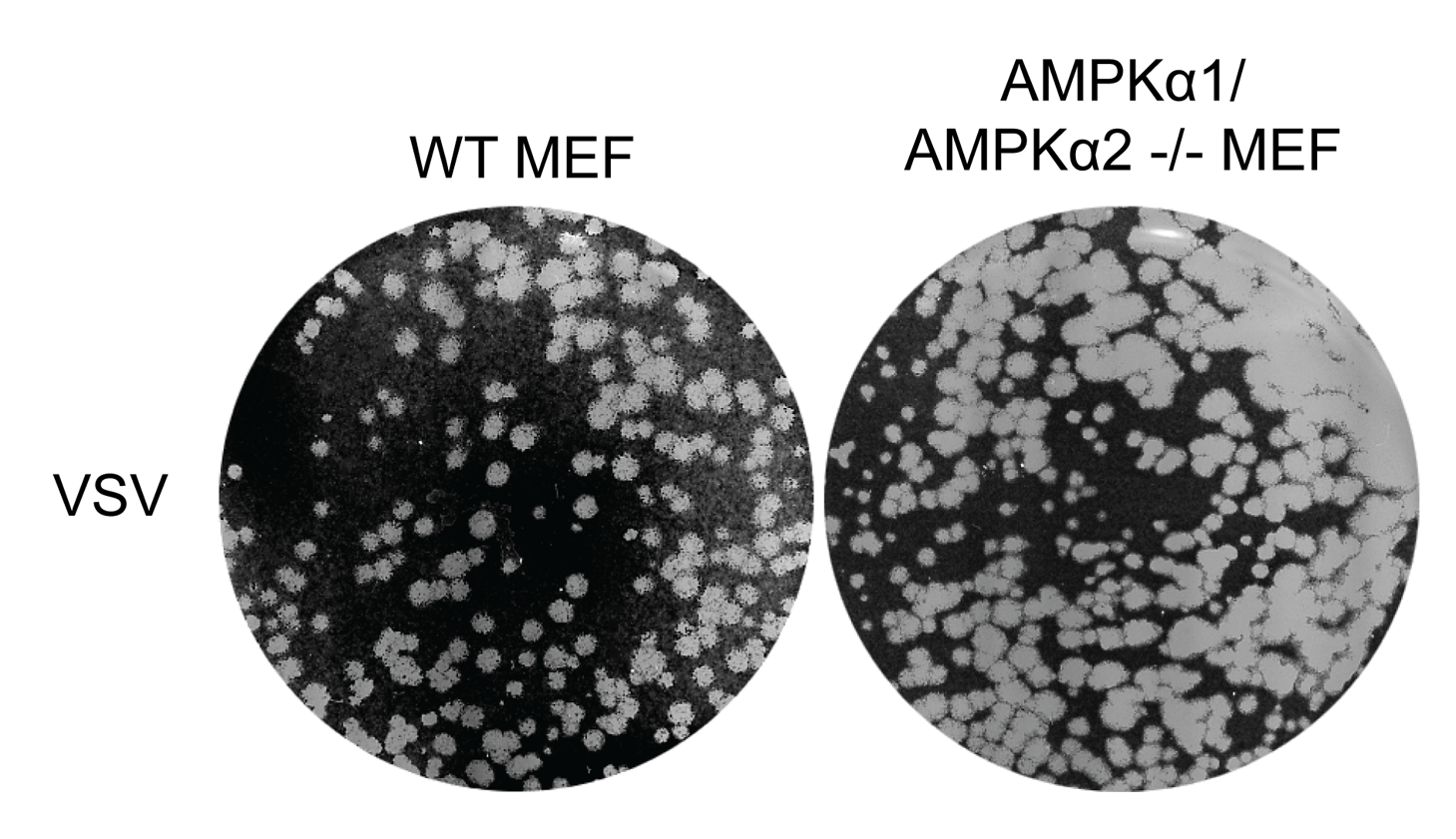

Supplement: Figure S7 — AMPK is not required for Vesicular Stomatitis Virus infection. Plaque assays were performed on wild type or AMPKα1/AMPKα2 −/− MEFs. There was no decrease in plaque number observed in the mutant cells. A representative experiment of three is shown. (1.01 MB TIF) [file ppat.1000954.s008.tif]

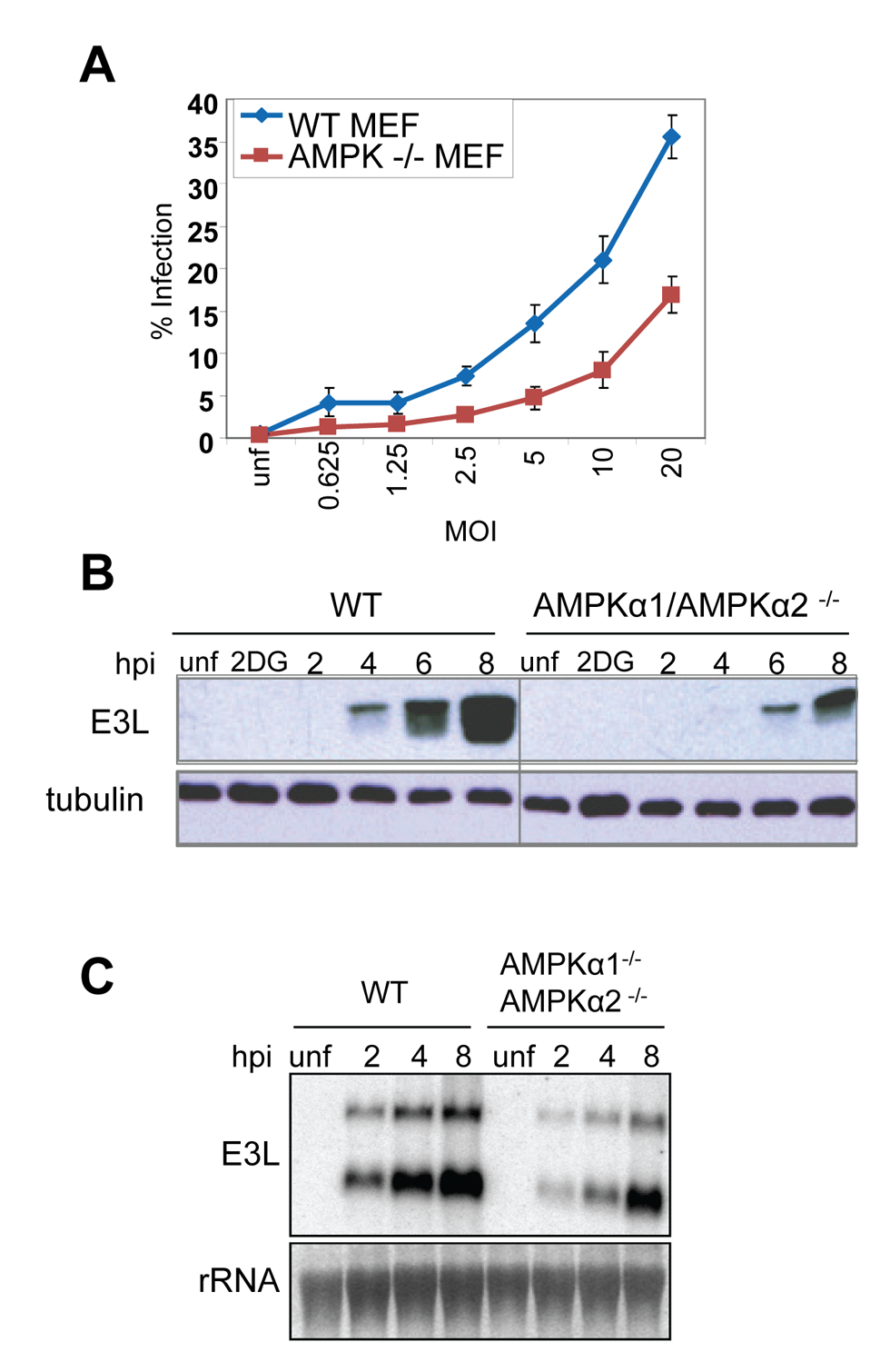

Supplement: Figure S8 — AMPK promotes early vaccinia infection in mammalian cells. A. Lack of AMPKα leads to decreased vaccinia infectivity in MEFs. Wild type or AMPKα1/AMPKα2 −/− MEFs were infected with the indicated MOI for 8 hours and processed for immunofluorescence. Data is displayed as average percentage of infected cells for a representative experiment. B. Loss of AMPKα leads to a decrease in viral mRNA production in AMPKα1/AMPKα2 −/− MEFs. Northern blot of viral mRNA levels in WT or AMPKα1/AMPKα2 −/− MEFs at indicated times post infection (MOI = 10). Blots were probed for virally encoded E3L or a ribosomal RNA loading control. C. Wild type or AMPKα1/AMPKα2 −/− cells were infected (MOI = 10) for the indicated times and probed for E3L by immunoblot. (0.51 MB TIF) [file ppat.1000954.s009.tif]

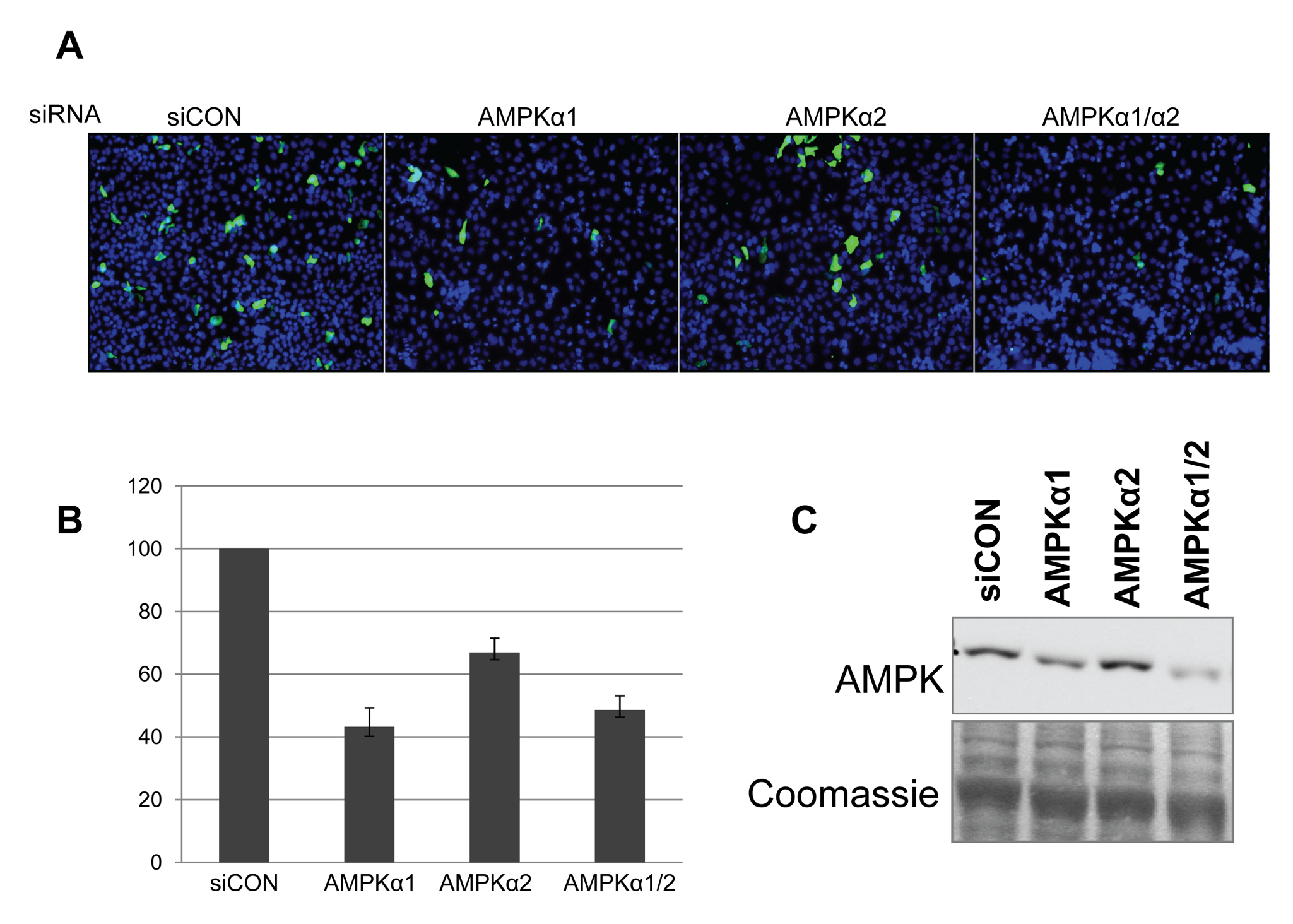

Supplement: Figure S9 — siRNA targeting AMPK inhibits vaccinia infection in mammalian cells. A. U2OS cells were treated with non-targeting siRNA (siCON) or siRNA targeting AMPKα1 or AMPKα2 and infected with vaccinia virus (MOI 10), and stained for E3L expression after 8 hours. B. Quantification of percent infection from A. The average of duplicate experiments; error bars represent mean of the percent infection for each experiment. C. Western blot probing total AMPKα after siRNA treatment. (1.69 MB TIF) [file ppat.1000954.s010.tif]

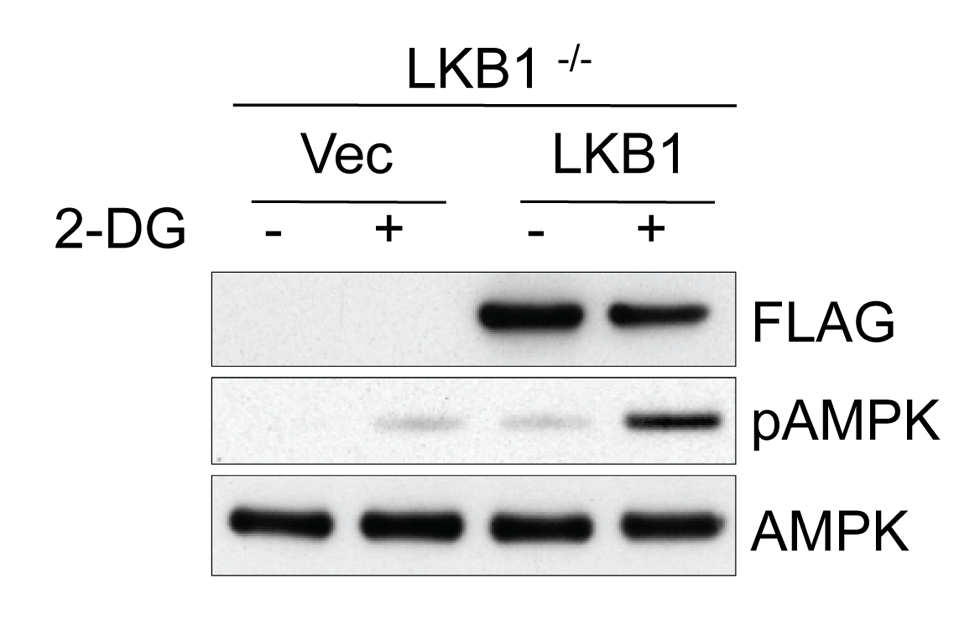

Supplement: Figure S10 — LKB1 cDNA rescues the LKB1 null cells. LKB1 −/− MEFs were complemented with a vector control (Vec) or FLAG-LKB1 (LKB1) cDNA and were mock treated, or treated with 2-deoxyglucose (2DG) which leads to LKB1-dependent AMPK phosphorylation for 30 min. Protein lysates were collected and probed by immunoblot for FLAG, phospho- or total-AMPKα expression. (0.72 MB TIF) [file ppat.1000954.s011.tif]

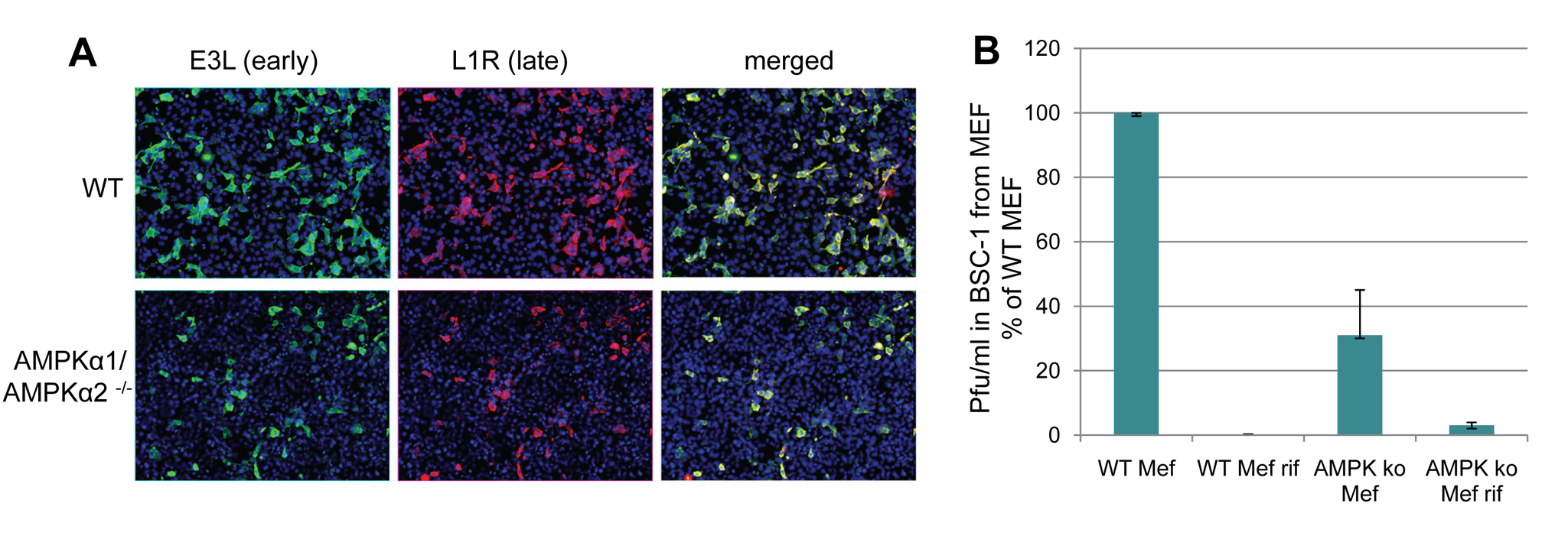

Supplement: Figure S11 — Vaccinia produced in AMPK deficient cells is infectious. A. AMPK is not required for late vaccina protein expression. WT and AMPKα1/AMPKα2 −/− MEFs infected with vaccinia for 8 hours were stained for early (E3L, green) and late (L1R, red) vaccinia protein expression. B. Infectious virus is produced in AMPK deficient cells. Vaccinia grown for 12 hours in WT and AMPKα1/AMPKα2 −/− MEFs was titered in BSC-1 cells. Rifampicin (Rif) was added as a control for detecting incoming virus. The relative pfu/ml in BSC-1 cells was graphed as the mean + standard deviation of triplicate experiments. The decrease in virus produced was similar to decrease in virus entry. (1.53 MB TIF) [file ppat.1000954.s012.tif]

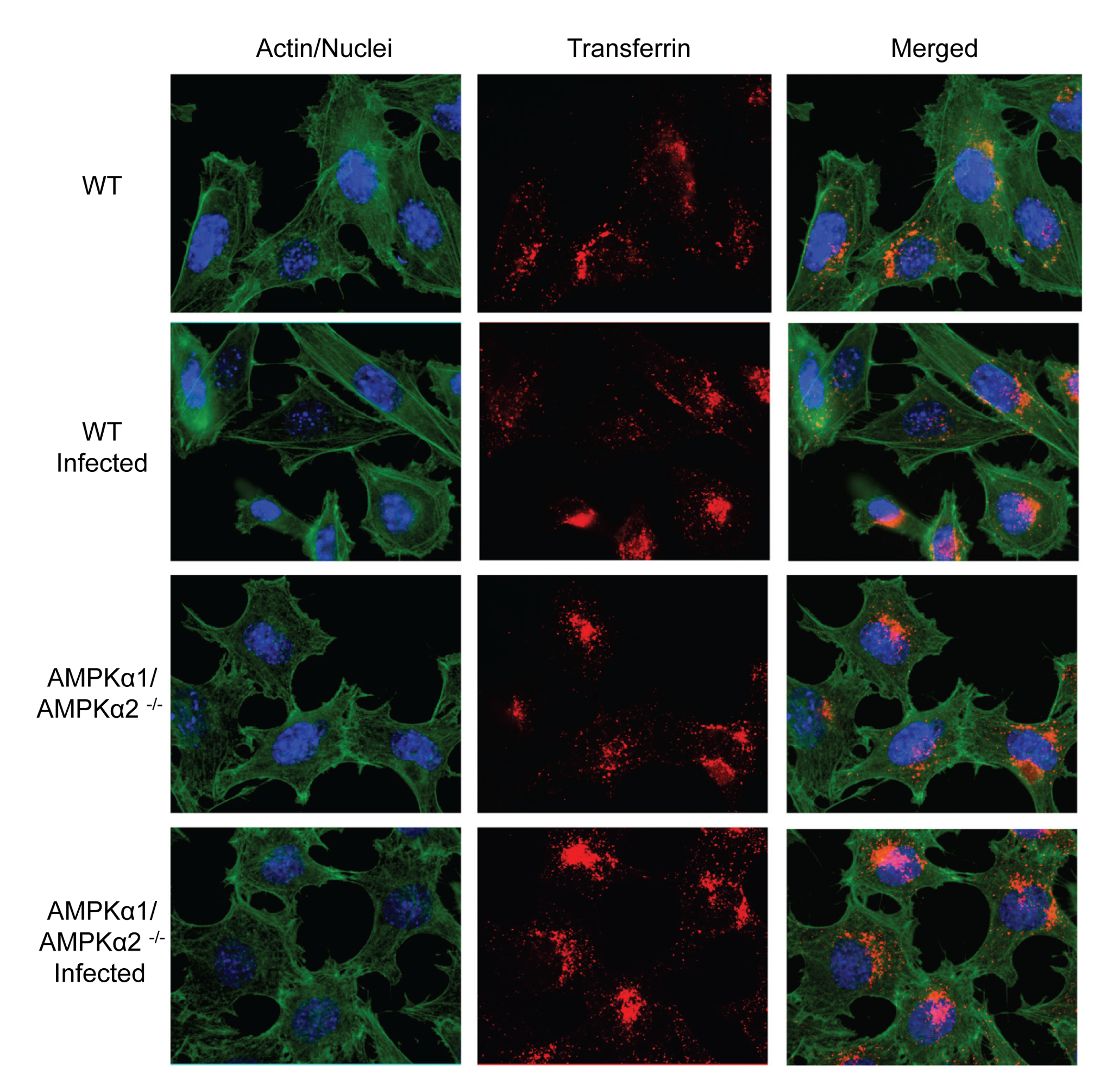

Supplement: Figure S12 — AMPK deficient cells undergo efficient receptor-mediated endocytosis. Transferrin uptake assays were performed in the presence or absence of virus. Wild type and AMPKα1/AMPKα2 −/− MEFs were either infected or mock-infected and treated with 594-transferrin (red), processed for microscopy, and stained tovisualize actin (phalloidin (green)) and nuclei (Hoescht 33342 (blue)). Representative images from triplicate experiments are shown. (3.74 MB TIF) [file ppat.1000954.s013.tif]

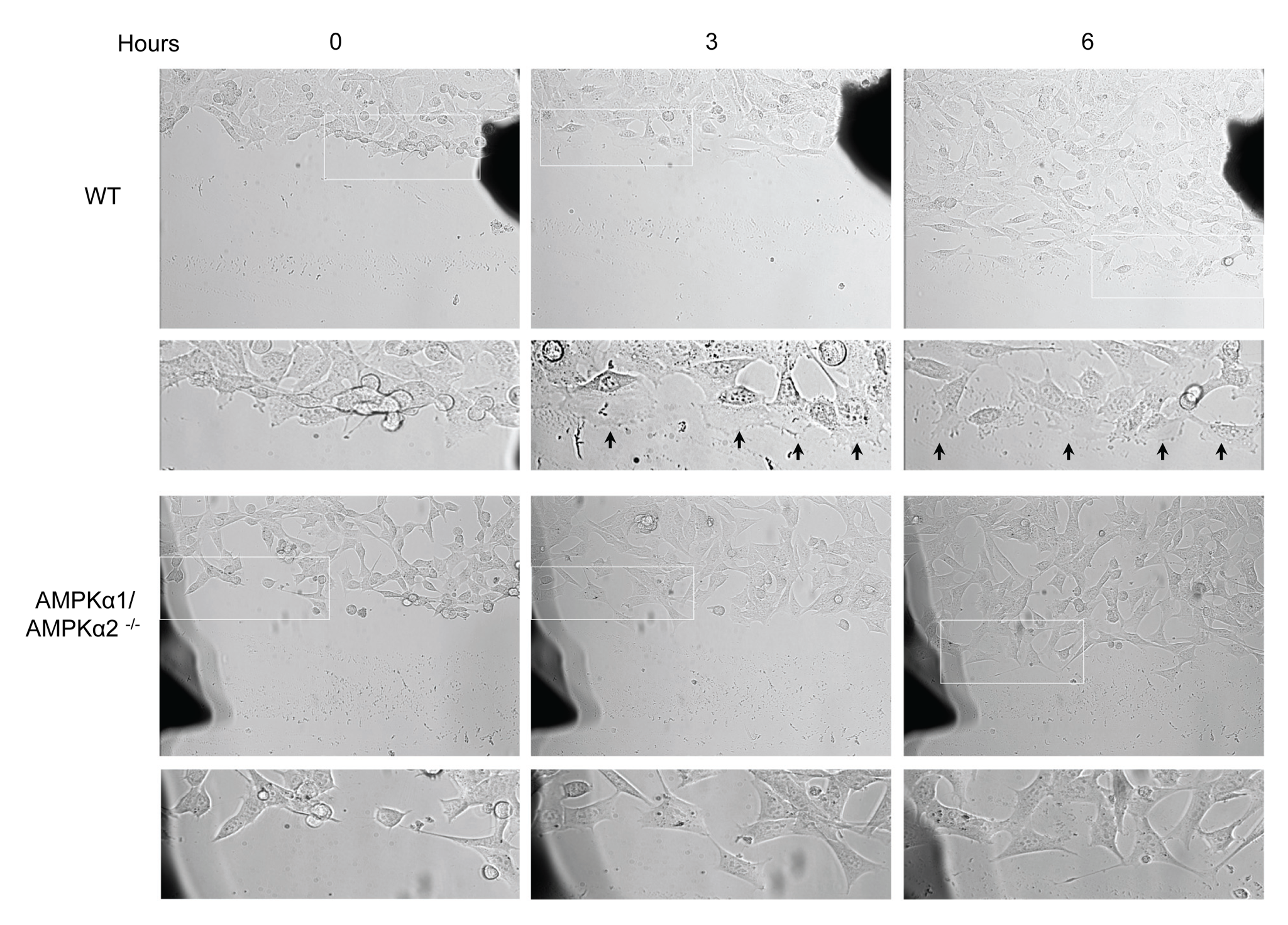

Supplement: Figure S13 — AMPK deficient cells are defective in lamellipodia formation during wound healing. A scratch was made in a confluent monolayer of wild type or AMPKα1/AMPKα2 −/− MEFs, and monitored over time. Images were taken using a 20X and 63X objective immediately after wounding (T = 0), and again after 3 and 6 hours to determine the morphology of the cells at the wound front. Polarized cells with lamellipodia are visible at the wound front of wild type MEFs (arrows). Representative images from triplicate experiments are shown. (3.43 MB TIF) [file ppat.1000954.s014.tif]

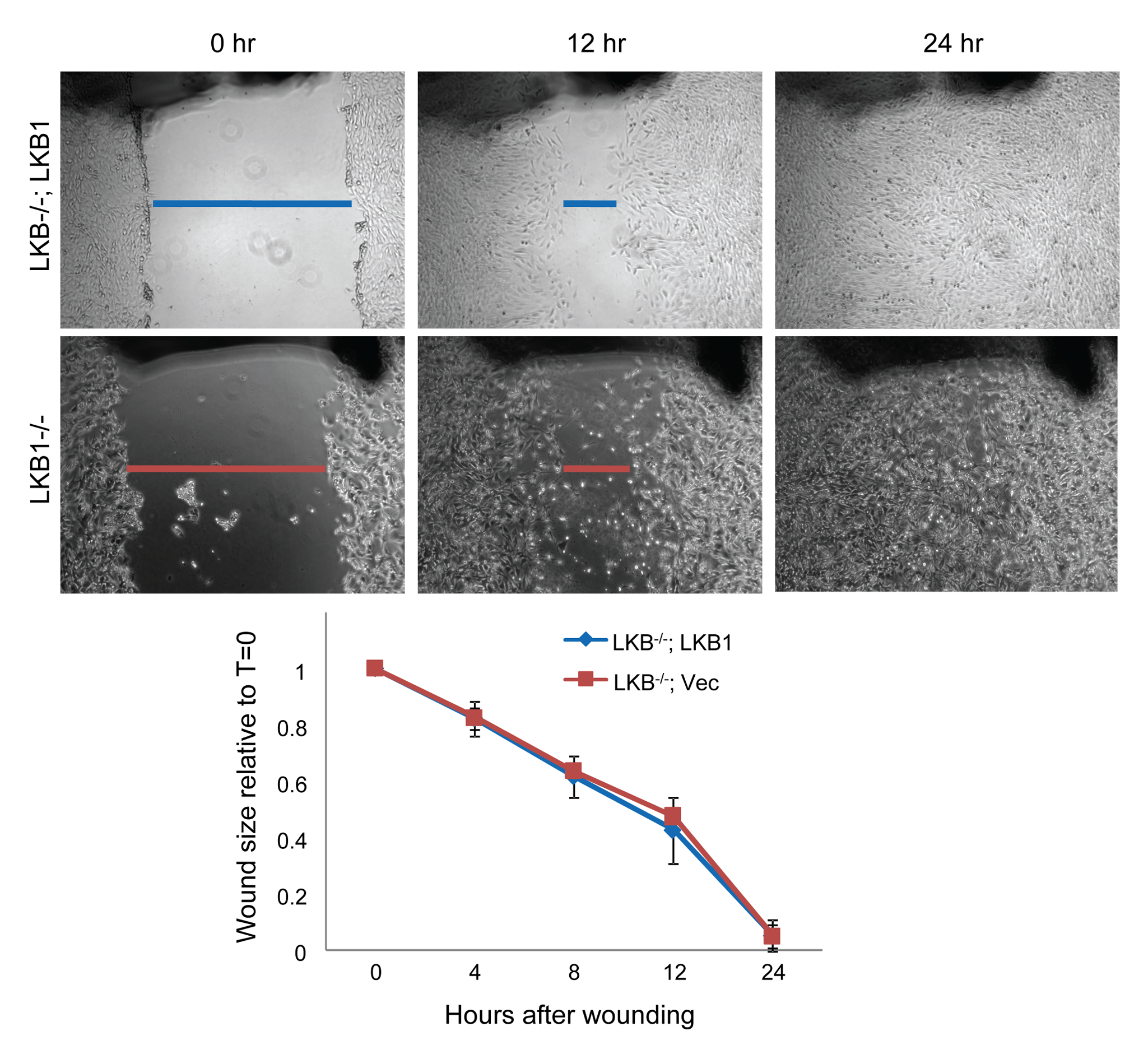

Supplement: Figure S14 — Cellular motility is LKB1-independent. Scratches were introduced into a confluent monolayer of LKB1 −/−, Vec or LKB1 −/−; LKB1 cDNA MEFs, and monitored over time for closure. Representative images from triplicate experiments are shown immediately after wounding (T = 0) and after 12 or 24 hours. The reduction in wound width is quantified over time. Data are normalized to initial would width at T = 0, and presented as means of three independent experiments with four wounds per set. (3.01 MB TIF) [file ppat.1000954.s015.tif]
